# Design, synthesis and antiproliferative activity of novel colchicine derivatives: selective inhibition of melanoma cell proliferation

**DOI:** 10.3389/fphar.2025.1528235

**Published:** 2025-06-27

**Authors:** Puneet Kumar, Tusharika Kotra, Waseem I. Lone, Yassir Arfath, Harshita Tiwari, Ashutosh Kumar Shukla, Zabeer Ahmed, Sheikh Rayees, Jasha Momo H. Anal

**Affiliations:** ^1^ Natural Products and Medicinal Chemistry Division, CSIR-Indian Institute of Integrative Medicine, Jammu, India; ^2^ Academy of Scientific and Innovative Research (AcSIR), Ghaziabad, India; ^3^ Pharmacology Division, CSIR-Indian Institute of Integrative Medicine, Jammu, India; ^4^ Drug Chemistry Research Centre, Indore, India

**Keywords:** *Colchicum autumnale*, colchicine, cancer, melanoma, proliferation, SRB assay

## Abstract

Colchicine binds to tubulin and destabilizes microtubules, stopping cell division and causing apoptosis. Its anti-cancer property affects microtubule integrity despite its reported toxicity. A series of novel colchicine derivatives were synthesized using the multi-component reaction method and evaluated for their antiproliferative properties, aiming to enhance their efficacy as anti-cancer agents compared to the parent compound, colchicine. With the SRB assay, we tested these derivatives for their anti-cancer efficacy against lung, breast, and melanoma using several human cancer cell lines, including A549, MCF-7, MDAMB-231, and A375. The study identified a derivative, **3g**, as notably more effective against melanoma cells, with a selectivity index about two times higher than colchicine. We further investigated the anti-cancer efficacy of compound **3g** on human melanoma cells using additional *in vitro* models, including the wound healing assay and colony formation assay. Compound **3g** inhibited colony formation by up to 62.5% and reduced the migration potential of melanoma cells by 69%. The *in silico* studies reveal the probable interactions with the colchicine binding sites that have a comparable pose to colchicine. **3g** formed a hydrogen bond with Cys241, Asn258 and a salt bridge with Lys352, which is important for its tubulin polymerization inhibitory activity. These findings suggest that **3g** could selectively target melanoma cells, minimizing toxicity to healthy cells and potentially providing a safer and more effective treatment option with improved therapeutic outcomes.

## 1 Introduction

Colchicine is a tropolone alkaloid natural product and an FDA-approved drug to treat gout and familial Mediterranean fever (Schlesinger et al., 2020). It is an antimitotic compound that binds to *β*-tubulin, destabilizes microtubules, and promotes depolymerization, leading to cell cycle arrest, apoptosis, and cell death (Ghawanmeh et al., 2018). Despite its ability to induce apoptosis, colchicine is not widely used as an anticancer drug, due to relatively high toxicity in the gastrointestinal tract, which is one of the three phases of toxicity that limit the use of certain compounds in cancer chemotherapy (Banerjee et al., 1997; Imazio et al., 2015; Tardif et al., 2022). Chemical modifications, derivatizations, and structure optimizations of colchicine at rings A, B, and C in colchicine result in functionality conversion and affect biological activity, which improved its therapeutic potential and safety profile (Singh et al., 2015; Dubey et al., 2017; Gracheva et al., 2020; Iqbal Lone et al., 2024). In recent years, a low-dose colchicine tablet branded as Lodoco was the first anti-inflammatory atheroprotective therapy for cardiovascular disease to reduce the risk of myocardial infarction, stroke, coronary revascularization, and cardiovascular death in adults (Banach and Penson, 2021; Halsey, 2023). Therefore, colchicine and its derivatives promise the potential of developing novel anti-cancer agents and therapeutic approaches (Kumar et al., 2017).

Cancer is a global public health challenge, marked by high prevalence, diverse types, and increasing incidence (Arnold et al., 2020). Advancing cancer drug discovery is imperative for improving treatments and reducing its impact on individuals and societies (Huang et al., 2021). Melanoma is an aggressive cancer with a high potential for metastasis. When it undergoes malignant transformation, it develops into one of the most lethal variants of skin cancer. While surgical excision is a frequently used successful treatment for early-stage melanomas, advanced cases have a grim prognosis, with an average survival duration of three to 11 months. Recent high-throughput genomic screening has shown the diverse character of melanoma by identifying several mutations driving its progression, and the cytotoxic effects and multidrug resistance linked to existing chemotherapeutic drugs restrict the treatment options for melanoma. Even though melanoma accounts for only 5% of all skin cancer cases, it is the cause of over 75% of skin cancer-related deaths, and the rate of incidence of melanoma is still on the rise (Smalley, 2010; Khan et al., 2023).

In this study, we synthesized novel colchicine derivatives using the Biginelli Multi-component Reaction (MCR) method, which has been vastly valuable for drug discovery (Kaur et al., 2017; Grazia Martina et al., 2023; Shahi et al., 2024) and screened them for antiproliferative activity against a panel of human cancer cell lines.

## 2 Experimental procedure

### 2.1 Extraction and isolation of colchicine and synthesis of colchicine aldehyde

**FIGURE 1:**
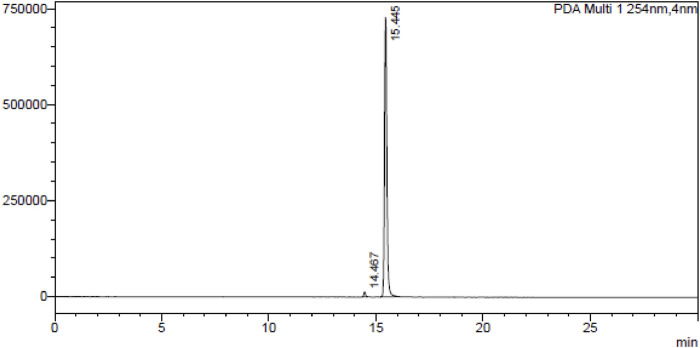
HPLC chromatogram of colchicine at λ254 nm.

A dried tuber (50.0 gm) of *Gloriosa superba* L. was extracted with MeOH: H_2_O (1:1) and colchicine was isolated by column chromatography using neutral alumina loaded with hexane. The eluting solvents chloroform and toluene in a ratio of 97:3. The fractions containing colchicine were collected, separated, and compared with standard colchicine, identified by thin layer chromatography (TLC); the fraction was evaporated in rotavaporatory and crystallized using chloroform as described in this procedure (Kannan et al., 2007). Purity of colchicine was determined by HPLC, as shown in Figure 1. Then, colchicine (1.0 mmol) was dissolved in dichloromethane (DCM, 25 mL) in a round-bottom flask, and dichloromethyl methyl ether (1.0 mmol) was added to the solution. The reaction mixture was stirred at 0°C for 30 min. After that, tin chloride (1.5 mmol) was added drop by drop to the reaction mixture, and stirring was continued at room temperature for 3.0 h. Once the reaction was complete (monitored by TLC), it was slowly quenched with ice water. The product was extracted with ethyl acetate, and the combined organic layer was dried with anhydrous sodium sulfate. The solution was concentrated using a rotary evaporator, and the residue was purified by silica gel column chromatography using hexane and ethyl acetate to obtain the pure product (Iqbal Lone et al., 2024).

### 2.2 Synthesis of colchicine derivatives

For the preparation of colchicine derivatives **3a** to **3j**, colchicine aldehyde **(2)** (1.0 mmol) and *β*-keto ester (2.0 mmol) were dissolved in ethanol (25 mL), taken in a round bottom flask after ammonia/urea/thiourea was added (2.0 mmol) dropwise. Then zinc chloride was added in a catalytic amount (0.3 mmol) and refluxed the mixture at 80°C for 5 h. After the reaction (product monitored by TLC) was completed, it was quenched slowly with sodium bicarbonate dissolved in water (for neutralizing acid). The pure product was obtained after column chromatography.

Compound **3a**: 4-((*R*)-7-acetamido-1,2,3,10-tetramethoxy-9-oxo-5,6,7,9-tetrahydrobenzo [a]heptalen-4-yl)-6-methyl-2-thioxo-1,2,3,4-tetrahydropyrimidine-5-carboxylate.

^1^H NMR (400 MHz, MeOD) δ 7.29–7.20 (m, 2H), 7.10 (d, *J* = 10.6 Hz, 1H), 4.38 (s, 1H), 3.93 (d, *J* = 4.4 Hz, 1H), 3.89 (d, *J* = 7.5 Hz, 3H), 3.81 (dd, *J* = 16.6, 8.2 Hz, 6H), 3.45 (d, *J* = 3.9 Hz, 3H), 2.16 (d, *J* = 16.6 Hz, 3H), 2.02 (dd, *J* = 17.5, 12.3 Hz, 1H), 1.90 (s, 3H), 1.72–1.62 (m, 1H), 1.19 (d, *J* = 7.4 Hz, 3H), 0.91–0.79 (m, 3H). ^13^C NMR (101 MHz, MeOD) δ 179.56, 171.32, 166.14, 164.18, 152.70, 151.02, 136.99, 136.41, 132.93, 129.33, 113.53, 60.23, 60.17, 60.07, 59.53, 55.65, 52.42, 34.87, 31.69, 30.76, 29.38, 29.10, 28.68, 24.63, 22.36, 21.05, 16.74, 13.19, 13.08. LC-MS (ESI) calcd for Chemical Formula: C_30_H_35_N_3_O_8_S 598.2223 and, found 598.2226.

Compound **3b**: Ethyl 4-((*R*)-7-acetamido-1,2,3,10-tetramethoxy-9-oxo-5,6,7,9-tetrahydrobenzo [a]heptalen-4-yl)-2-imino-6-methyl-1,2,3,4-tetrahydropyrimidine-5-carboxylate.

^1^H NMR (400 MHz, MeOD) δ 7.28–7.21 (m, 2H), 7.09 (dd, *J* = 11.0, 4.4 Hz, 1H), 4.37 (dd, *J* = 11.8, 6.0 Hz, 1H), 3.89 (d, *J* = 8.3 Hz, 3H), 3.81 (d, *J* = 4.4 Hz, 5H), 3.44 (d, *J* = 7.4 Hz, 3H), 2.18 (s, 3H), 2.07–1.96 (m, 1H), 1.90 (d, *J* = 3.0 Hz, 3H), 1.69 (dd, *J* = 11.7, 5.8 Hz, 1H), 1.19 (s, 1H), 0.90 (dt, *J* = 31.2, 7.0 Hz, 3H). ^13^C NMR (101 MHz, MeOD) δ 179.57, 171.32, 164.16, 152.67, 150.69, 129.70, 129.31, 129.18, 113.54, 60.19, 60.02, 59.34, 59.31, 55.63, 52.41, 52.34, 34.95, 29.39, 24.56, 21.01, 20.98, 17.24, 13.21. LC-MS (ESI) calcd for Chemical Formula: C_30_H_36_N_4_O_8_ Exact Mass: 581.25, found 582.22.

Compound **3c**: phenyl 4-((R)-7-acetamido-1,2,3,10-tetramethoxy-9-oxo-5,6,7,9-tetrahydrobenzo [a]heptalen-4-yl)-6-methyl-2-oxo-1,2,3,4-tetrahydropyrimidine-5-carboxylate.

^1^H NMR (400 MHz, MeOD) *δ* 7.25–7.19 (m, 1H), 7.10 (d, *J* = 2.5 Hz, 2H), 7.02 (s, 2H), 6.91–6.81 (m, 2H), 5.00 (dd, *J* = 26.7, 12.5 Hz, 1H), 4.68 (d, *J* = 12.2 Hz, 1H), 4.31 (dd, *J* = 11.7, 6.3 Hz, 1H), 3.92 (s, 3H), 3.75 (d, *J* = 12.0 Hz, 5H), 3.44 (s, 3H), 2.88 (dd, *J* = 14.0, 4.8 Hz, 1H), 2.19 (d, *J* = 18.6 Hz, 3H), 2.09 (s, 1H), 1.84 (d, *J* = 3.8 Hz, 3H), 1.55–1.39 (m, 1H). ^13^C NMR (101 MHz, MeOD) δ 179.54, 171.28, 166.05, 164.10, 150.63, 136.50, 136.29, 129.18, 128.04, 127.51, 113.51, 65.23, 60.22, 60.02, 55.70, 52.39, 34.98, 24.12, 21.04, 20.97, 17.37. LC-MS (ESI) calcd for Chemical Formula: C_34_H_35_N_3_O_9_ Exact Mass: 629.24 and, found 644.30 [M+Na]^+^.

Compound **3d**: Ethyl 4-((*R*)-7-acetamido-1,2,3,10-tetramethoxy-9-oxo-5,6,7,9-tetrahydrobenzo [a]heptalen-4-yl)-2-oxo-6-(trichloromethyl)-1,2,3,4-tetrahydropyrimidine-5-carboxylate.

^1^H NMR (400 MHz, MeOD) *δ* 7.23 (ddd, *J* = 23.2, 13.9, 7.6 Hz, 2H), 7.10 (d, *J* = 10.9 Hz, 1H), 4.40–4.27 (m, 1H), 4.12–3.99 (m, 1H), 3.96–3.92 (m, 2H), 3.91 (s, 3H), 3.89–3.79 (m, 6H), 3.51 (s, 1H), 3.47 (q, *J* = 4.5 Hz, 3H), 2.23–2.00 (m, 2H), 1.92 (d, *J* = 2.1 Hz, 3H), 1.81–1.70 (m, 1H), 1.22–1.11 (m, 3H), 1.01–0.67 (m, 2H). ^13^C NMR (101 MHz, MeOD) δ 184.25, 179.57, 171.37, 164.40, 164.30, 164.26, 153.73, 152.29, 151.82, 136.61, 136.55, 136.49, 136.37, 129.38, 129.31, 113.43, 88.61, 60.40, 60.33, 60.30, 60.22, 60.16, 55.68, 52.16, 51.73, 35.04, 29.37, 21.01, 12.97, 12.61. LC-MS (ESI) calcd for Chemical Formula: C_30_H_32_Cl_3_N_3_O_9_ Exact Mass: 683.12 and, found 704.20 [M+Na]^+^.

Compound **3e**: Ethyl 4-((*R*)-7-acetamido-1,2,3,10-tetramethoxy-9-oxo-5,6,7,9-tetrahydrobenzo [a]heptalen-4-yl)-6-(2,6-dichloro-5-fluoropyridin-3-yl)-2-oxo-1,2,3,4-tetrahydropyrimidine-5-carboxylate.

^1^H NMR (400 MHz, MeOD) *δ* 7.71 (ddd, *J* = 20.5, 10.9, 6.7 Hz, 1H), 7.29–7.23 (m, 2H), 7.10 (d, *J* = 11.0 Hz, 1H), 4.38 (dd, *J* = 11.6, 6.0 Hz, 1H), 3.96 (dd, *J* = 17.5, 8.2 Hz, 3H), 3.91 (s, 3H), 3.86 (d, *J* = 6.4 Hz, 2H), 3.81 (s, 1H), 3.78–3.70 (m, 1H), 3.62 (ddd, *J* = 13.9, 7.0, 4.0 Hz, 1H), 3.47 (t, *J* = 4.1 Hz, 3H), 2.09 (dd, *J* = 15.1, 5.2 Hz, 1H), 1.90 (s, 3H), 1.74 (dd, *J* = 17.0, 8.0 Hz, 1H), 1.19 (s, 1H), 0.72–0.60 (m, 3H). ^13^C NMR (101 MHz, MeOD) δ 179.58, 175.01, 171.33, 164.20, 129.28, 119.17, 116.70, 113.49, 110.59, 60.51, 60.21, 60.16, 60.12, 60.07, 59.92, 58.84, 55.63, 52.43, 52.36, 52.32, 31.15, 29.37, 20.99, 20.65, 12.70. LC-MS (ESI) calcd for Chemical Formula: C_34_H_33_C_l2_FN_4_O_9_ Exact Mass: 731.1687 and, found 731.1685.

Compound **3f**: Ethyl 4-((*R*)-7-acetamido-1,2,3,10-tetramethoxy-9-oxo-5,6,7,9-tetrahydrobenzo [a]heptalen-4-yl)-2-oxo-6-(perfluoroethyl)-1,2,3,4-tetrahydropyrimidine-5-carboxylate.

^1^H NMR (400 MHz, MeOD) δ 7.26 (s, 2H), 7.21 (d, *J* = 10.7 Hz, 2H), 7.10 (d, *J* = 11.1 Hz, 3H), 5.17 (d, *J* = 11.2 Hz, 2H), 4.31–4.22 (m, 4H), 3.92–3.89 (m, 15H), 3.87 (d, *J* = 3.7 Hz, 7H), 3.83–3.75 (m, 10H), 3.47 (s, 6H), 1.91 (s, 8H), 1.19 (s, 4H), 0.83 (t, *J* = 7.1 Hz, 7H). ^13^C NMR (101 MHz, MeOD) δ 179.57, 171.18, 169.77, 164.31, 154.65, 152.23, 151.92, 145.88, 136.48, 134.05, 129.44, 129.38, 121.54, 113.43, 61.06, 60.38, 60.26, 60.24, 55.67, 53.42, 52.09, 48.88, 34.71, 25.80, 24.97, 20.95, 13.03. LC-MS (ESI) calcd for Chemical Formula: C_31_H_32_F_5_N_3_O_9_ Exact Mass: 685.21 and, found 704.20 [M+Na]^+^.

Compound **3g**: Ethyl 4-((*S*)-7-acetamido-1,2,3,10-tetramethoxy-9-oxo-5,6,7,9-tetrahydrobenzo [a]heptalen-4-yl)-2-oxo-6-(perfluoroethyl)-1,2,3,4-tetrahydropyrimidine-5-carboxylate.

^1^H NMR (400 MHz, MeOD) δ 7.26 (s, 1H), 7.22 (d, *J* = 10.8 Hz, 1H), 7.10 (d, *J* = 11.0 Hz, 1H), 4.37 (dd, *J* = 11.9, 6.3 Hz, 1H), 4.03 (d, *J* = 11.2 Hz, 1H), 3.93 (s, 2H), 3.91 (s, 2H), 3.86 (s, 1H), 3.83 (s, 2H), 3.82 (s, 1H), 3.46 (d, *J* = 1.7 Hz, 3H), 1.94 (s, 1H), 1.91 (s, 2H), 1.84 (s, 1H), 1.19 (s, 1H), 0.77 (d, *J* = 7.1 Hz, 2H). ^13^C NMR (101 MHz, MeOD) δ 179.57, 171.38, 169.11, 164.26, 154.53, 152.31, 151.79, 146.18, 136.53, 136.31, 133.99, 129.47, 129.42, 121.91, 113.42, 60.80, 60.20, 60.13, 55.68, 55.66, 52.18, 35.06, 20.98, 12.67. LC-MS (ESI) calcd for Chemical Formula: C_31_H_32_F_5_N_3_O_9_ Exact Mass: 685.21 and, found 704.20 [M+Na]^+^.

Compound **3h**: ethyl 4-((*R*)-7-acetamido-1,2,3,10-tetramethoxy-9-oxo-5,6,7,9-tetrahydrobenzo [a]heptalen-4-yl)-6-ethoxy-2-oxo-1,2,3,4-tetrahydropyrimidine-5-carboxylate.

^1^H NMR (400 MHz, MeOD) δ 7.26 (s, 1H), 7.18 (d, *J* = 10.8 Hz, 1H), 7.09 (d, *J* = 11.1 Hz, 1H), 5.84 (d, *J* = 11.3 Hz, 1H), 4.27 (dd, *J* = 12.0, 6.5 Hz, 1H), 4.17–4.08 (m, 2H), 4.04 (s, 3H), 4.01 (d, *J* = 11.3 Hz, 1H), 3.90 (s, 3H), 3.76 (ddd, *J* = 17.9, 12.5, 7.1 Hz, 2H), 3.45 (s, 3H), 2.44–2.32 (m, 1H), 1.97 (dd, *J* = 13.9, 6.2 Hz, 1H), 1.90 (s, 3H), 1.70 (td, *J* = 12.1, 5.5 Hz, 1H), 1.18 (t, *J* = 7.1 Hz, 3H), 0.81 (t, *J* = 7.1 Hz, 3H). ^13^C NMR (101 MHz, MeOD) δ 179.59, 171.42, 167.37, 166.94, 164.17, 159.21, 153.03, 152.61, 151.09, 144.85, 136.97, 136.10, 132.80, 129.41, 128.89, 124.62, 113.46, 78.08, 61.45, 61.04, 60.22, 60.17, 56.98, 55.62, 52.34, 35.01, 25.11, 21.01, 13.02, 12.76. LC-MS (ESI) calcd for Chemical Formula: C_31_H_37_N_3_O_10_ Exact Mass: 611.25 and, found 630 [M+NH_4_]^+^.

Compound **3i**: *N*-((7*R*)-4-(5-(cyclopropanecarbonyl)-6-methoxy-2-oxo-1,2,3,4-tetrahydro pyrimidin-4-yl)-1,2,3,10-tetramethoxy-9-oxo-5,6,7,9-tetrahydrobenzo [a]heptalen-7-yl) acetamide.

^1^H NMR (400 MHz, MeOD) δ 7.29–7.22 (m, 2H), 7.09 (dd, *J* = 10.7, 5.7 Hz, 1H), 5.75 (s, 1H), 4.38 (dd, *J* = 9.0, 4.9 Hz, 1H), 3.89 (d, *J* = 11.4 Hz, 3H), 3.82 (d, *J* = 10.6 Hz, 6H), 3.45 (d, *J* = 9.3 Hz, 3H), 3.40 (d, *J* = 17.8 Hz, 3H), 2.83–2.61 (m, 1H), 2.32–2.16 (m, 1H), 2.02 (td, *J* = 14.1, 6.0 Hz, 1H), 1.90 (d, *J* = 10.2 Hz, 3H), 1.77–1.63 (m, 1H), 1.19 (s, 1H), 0.84 (d, *J* = 8.4 Hz, 3H). ^13^C NMR (101 MHz, MeOD) δ 179.58, 171.34, 171.30, 167.08, 164.17, 164.15, 152.75, 152.59, 150.72, 150.65, 145.43, 136.37, 132.72, 129.39, 129.33, 129.22, 113.55, 60.18, 60.02, 59.94, 55.63, 52.41, 52.22, 49.93, 49.91, 35.01, 24.17, 21.03, 21.01, 11.36 LC-MS (ESI) calcd for Chemical Formula: C_31_H_35_N_3_O_9_ Exact Mass: 594.2452 and, found 594.2453.

Compound **3j**: *N*-((7*S*)-4-(5-(cyclopropanecarbonyl)-6-methoxy-2-oxo-1,2,3,4-tetrahydro pyrimidin-4-yl)-1,2,3,10-tetramethoxy-9-oxo-5,6,7,9-tetrahydrobenzo [a]heptalen-7-yl) acetamide.

^1^H NMR (400 MHz, MeOD) δ 7.29–7.23 (m, 2H), 7.10 (dd, *J* = 11.1, 4.5 Hz, 1H), 4.38 (dd, *J* = 11.9, 6.1 Hz, 1H), 3.90 (d, *J* = 9.4 Hz, 3H), 3.84 (s, 3H), 3.80 (d, *J* = 5.3 Hz, 3H), 3.55 (dd, *J* = 12.4, 5.5 Hz, 2H), 3.45 (d, *J* = 3.4 Hz, 3H), 3.39 (d, *J* = 16.3 Hz, 3H), 2.74–2.65 (m, 2H), 1.99 (dd, *J* = 14.5, 7.3 Hz, 3H), 1.91 (d, *J* = 4.0 Hz, 3H), 1.69 (td, *J* = 11.9, 6.0 Hz, 1H), 1.19 (s, 1H). ^13^C NMR (101 MHz, MeOD) δ 179.57, 171.35, 166.40, 164.14, 152.59, 150.78, 150.64, 136.48, 132.81, 129.48, 129.25, 129.23, 113.57, 60.23, 60.16, 60.05, 60.02, 55.63, 52.41, 52.24, 49.92, 43.76, 43.68, 31.16, 28.92, 24.20, 21.03. LC-MS (ESI) calcd for Chemical Formula: C_31_H_35_N_3_O_9_ Exact Mass: 594.2452 and, found 594.2453.

### 2.3 Antiproliferative study

#### 2.3.1 Cell culture and cell lines

Roswell Park Memorial Institute (RPMI)-1640 medium, Dulbecco’s modified eagle medium high glucose (DMEM; Gibco) supplemented with 10% Fetal Bovine Serum (FBS; Gibco, and 1% antibiotic-antimycotic (Gibco; Anti-Anti 100x), phosphate-buffered saline (PBS; Gibco), 0.25% trypsin (Gibco), sodium pyruvate (Sigma-Aldrich), HEPES (Gibco). Human cancer cell lines A549 (lung carcinoma), MCF-7 (breast carcinoma), MDAMB-231 (breast carcinoma), A375 (melanoma), and normal human embryonic kidney cells (HEK-293) were taken from Dr. Sheikh Tasduq Abdullah and Dr. Boobalan Gopu (CSIR-IIIM).

#### 2.3.2 *In vitro* cytotoxicity

##### 2.3.2.1 Sulforhodamine B (SRB) assay

Sulforhodamine B (SRB), a semi-automated colorimetric assay, was used to evaluate the anti-cancerous properties of indicated colchicine derivatives. Their activity was tested against human cancer cell lines A549 (lung carcinoma), MCF-7 (breast carcinoma), MDAMB-231 (breast carcinoma), and A375 (melanoma). The selectivity index (SI) was calculated against normal human embryonic kidney cells (HEK-293) to determine cytotoxic selectivity. The cells (7 × 10^3^ cells/well) were seeded on 96 well plates, followed by overnight incubation. Multiple concentrations (50 µM, 20 µM, 10 µM, 5 µM, and 1 µM) of compounds were used to treat the cells, except for control wells that contained only cells with media. After 48 h of incubation, the cells attached to wells were fixed with 50 µL of chilled 50% TCA, and the plate was kept at 4°C for 1 h, as described previously (Malik et al., 2023; Kumar et al., 2024). The plate was washed (3–4 times) with distilled water (250 µL/well) and air-dried for 24 h. The next day, 100 µL of 0.4% SRB dye was added to each well, and the cells were incubated at room temperature for 1 h. The plate contents were discarded and washed 3 times with 1% glacial acetic acid (250 µL/well), followed by one last wash with distilled water and air drying for another 24 h. Subsequently, 100 µL of Tris base (10 mM, pH 10.5) was added to each well to dissolve the dye bound to fixed cells. The dissolved dye indicating growth inhibition was quantified by calculating optical density at 540 nm *via* microplate reader (Tecan infinite M200PRO, Switzerland). The IC_50_ values were calculated using GraphPad Prism Software Version 5.0. A favorable Selectivity Index (SI) is the most relevant parameter to detect anti-cancer potential *in vitro* as it indicates drug safety against cancer cells versus normal cells, efficacy of the drug, and selectivity. The selectivity index is calculated by using the formula.

$$\text{Selective}\;\text{Index}=\frac{{IC}_{50}\;\text{in}\;\text{Normal}\;\text{cells}}{{IC}_{50}\;\text{in}\;\text{Cancerous}\;\text{cell}\;\text{lines}}$$

##### 2.3.2.2 MTT assay

Cell viability was assessed using the MTT assay, as outlined in previous studies (Majeed et al., 2014; Amin et al., 2025). Briefly, A549, A375, MCF-7 and MDA-MB231 cells (10 × 10^3^ cells/well) were seeded in 96-well plates and incubated for 24 h at 37°C in a 5% CO_2_ atmosphere. After this incubation, the cells were treated with test compounds for another 48 h. At 44^th^ hour of treatment, the cells were incubated with MTT (2.5 mg/mL) for additional 4 h. At 48^th^ hour, DMSO was added (100 µL/well), to dissolve formazan crystals. Absorbance was measured at 570 nm. Cell viability was calculated relative to untreated controls. IC_50_ values were determined from independent triplicate experiments using GraphPad™ Prism 8.0 software.

#### 2.3.3 Colony formation assay

A colony formation assay was performed to assess the ability of **3g** to inhibit the reproductive and colony forming ability of A375 cells. The cells were seeded into a 6-well plate with a 1 × 10^3^ cells/well density. Next day, the cells were treated with multiple concentrations of **3g**, such as 30 µM, 20 µM, 10 µM and 1 µM, in fresh media for 48 h. The media was changed on alternate days till day 10, till untreated (negative control) became confluent. On day 10, the cells were washed with PBS, and colonies were observed under a microscope. The cells/colonies were fixed with 100% methanol for 20 min at 4°C and stained with 0.5% solution of crystal violet for 30 min at room temperature, as described previously (Kumar et al., 2024). Subsequently, the dye was removed from plates, and excess dye was rinsed by washing it with distilled water (3–5 times), followed by air drying. The colonies were counted using ImageJ software, and photographs were taken with a digital camera. The percentage of colonies formed was calculated using the formula mentioned below.

$$\text{Colony}\;\text{formation}\;\text{rate}\;\text{(\%)}=\frac{\text{Number}\;\text{of}\;\text{colonies}\;\text{in}\;\text{treatment}\;\text{group}}{\text{Number}\;\text{of}\;\text{colonies}\;\text{in}\;\text{control}\;\text{group}}\times 100$$

#### 2.3.4 Wound healing assay

Cell scratch assay, also known as wound healing assay, was performed to evaluate the impact of **3g** on migration of A375 cells. The cells were seeded into the 6-well plate with 4 × 10^3^ cells/well density. The next day, in fresh media, a horizontal scratch was administered in the 90% confluent monolayer using a sterile 200 µL tip, as described previously, with slight modification (Kumar et al., 2024). The cells were incubated for 48 h with multiple concentrations of **3g** such as 30 µM, 20 µM, 10 µM and 1 µM. Wound healing was observed using a Digital Inverted Fluorescence Microscope (Life Technologies EVOS FLc). The percentage of wound healing was calculated as.

$$\text{Wound}\;\text{Closure}\;\text{(\%)}=\frac{\text{Wound}\;\text{area}\;\text{at}\;0.0\;\text{hr}}{\text{Wound}\;\text{area}\;\text{at}\;48.0\;\text{hrs}}\times 100$$

#### 2.3.5 Molecular docking experiment

Before molecular docking studies, colchicine and its derivative, **3g**, underwent preparation using the OpenBabel module within PyRx software (O’Boyle et al., 2011). This process involved structural minimization via the universal force field (UFF) and subsequent conversion to PDBQT format, rendering them suitable for docking studies. The 3D structure of the tubulin target protein co-crystalized with the drug colchicine (PDB ID: 1SA0) was downloaded from the protein data bank. The structure was subsequently prepared using the Chimera DockPrep module by removing co-crystallized water, adding hydrogen atoms, and optimizing the pKa states (Pettersen et al., 2004). Chain B of the protein was used for molecular docking. The grid was generated around the drug colchicine at coordinates X = 117.219, Y = 90.179, and Z = 6.289 Å as described (Moussaoui et al., 2024). Molecular docking was performed using the AutoDock Vina module. The docking protocol was verified by redocking the co-crystalized colchicine to its binding site and comparing the root mean square deviation with the crystallized pose. After docking, the binding poses were visualized with the free Maestro visualizer.

#### 2.3.6 Pharmacokinetic property prediction

Pharmacokinetic (PK) properties, including absorption, distribution, metabolism, excretion, and toxicity (ADMET), are critical for drug development. In this study, pkCSM web-based platform (https://biosig.lab.uq.edu.au/pkcsm/) was used. For the prediction of ADMET properties. pkCSM uses graph-based signatures and machine learning algorithms to predict pharmacokinetic properties. It provides predictions for key ADMET parameters, such as water solubility, intestinal absorption, blood-brain barrier permeability, CYP450 interactions, and toxicity endpoints. For prediction of human liver microsomal stability PredMS (https://predms.netlify.app.), a random forest model based tool was used.

## 3 Results and discussion

### 3.1 Chemistry

**SCHEME 1:**
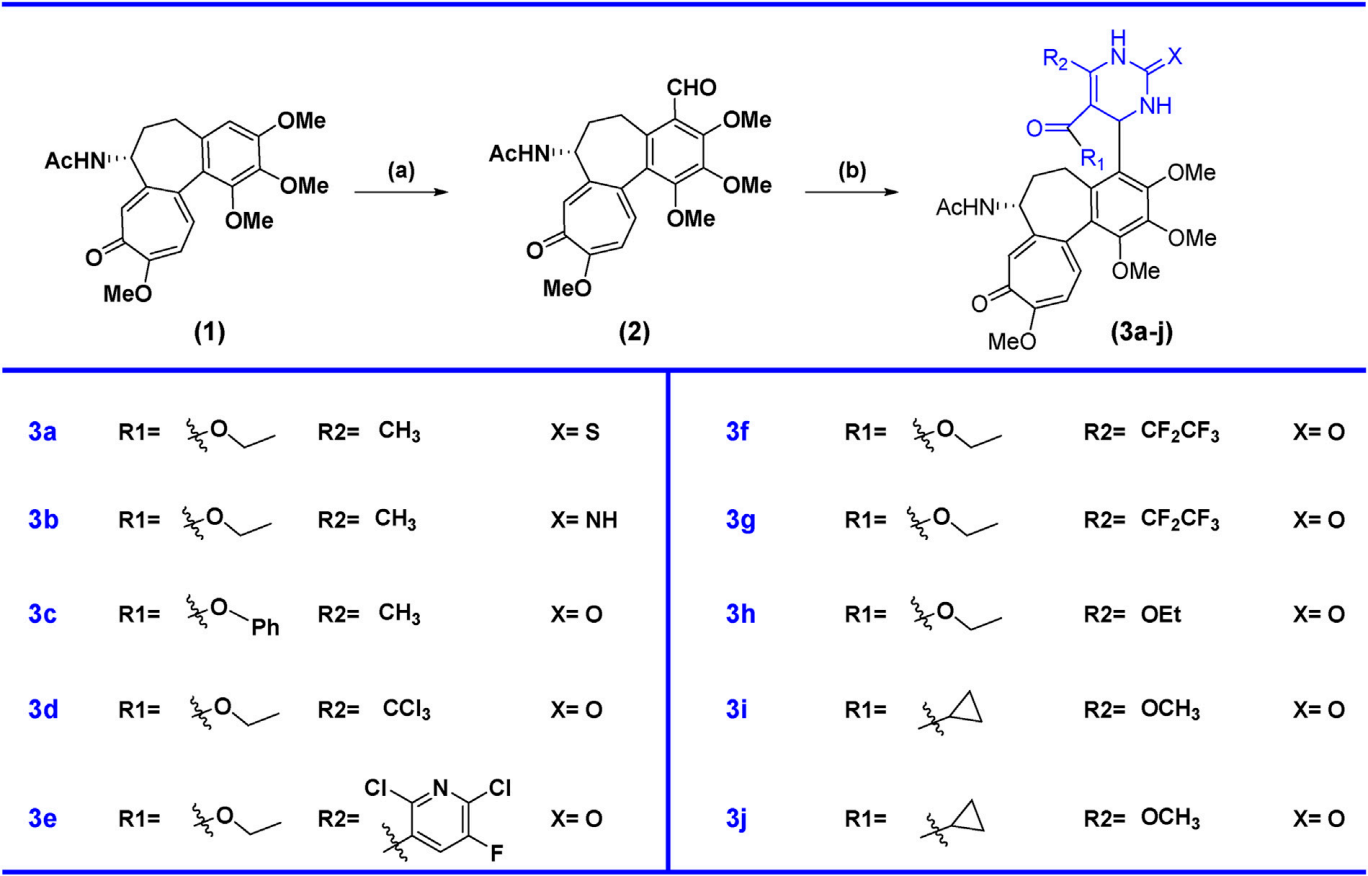
Synthesis of colchicine derivatives. Reagents and condition: **(a)** Dichloromethyl methyl ether (1.0 mmol), SnCl_2_ (1.5 mmol), DCM, 0°C, 70%; **(b)** β-keto ester (2.0 mmol), guanidine/urea/thiourea (2.0 mmol), ZnCl_2_ (cat), ethanol, 80°C, 10 h, 50%–60%.

Colchicine is a tricyclic system containing one six-membered and two seven-membered rings with an (*R*)-stereogenic center at the C-7 position. First, colchicine **(1)** was converted into colchicine aldehyde **(2)** at the C-4 position by using the dichloromethylmethyl ether, which acts as a formylating agent to formylate aromatic ring in the presence of Lewis acid tin tetrachloride at 0°C to obtained colchicine aldehyde (Scheme 1a). Further, we have synthesized colchicine derivatives using the Biginelli multi-component chemical reaction method to synthesize dihydropyrimidones. This multi-component reaction requires the colchicine aldehyde, *β*-keto ester, and urea/thiourea/guanidine (Scheme 1b). For the synthesis **3a**, we used thiourea with ethyl acetoacetate as a *β*-keto ester. Guanidine and ethyl acetoacetate as *β*-keto ester were used to synthesize **3b**. The rest of the compounds (**3c, 3d, 3e, 3f, 3g, 3h, 3i, and 3j**) were synthesized using urea and different *β*-keto ester in the presence of ZnCl_2_ catalytic amounts. We observed diastereomers in the case of compounds **3f** and **3g** and **3i** and **3j**, which were separated by HPLC, as shown in **3f**, **3g**, **3i**, and **3j**. In other compounds, we have only found single stereoisomers and other stereoisomers in a trace, so we only isolated the single compounds **3a, 3b, 3c, 3d, 3e, and 3h**.

### 3.2 Biology

**FIGURE 2:**
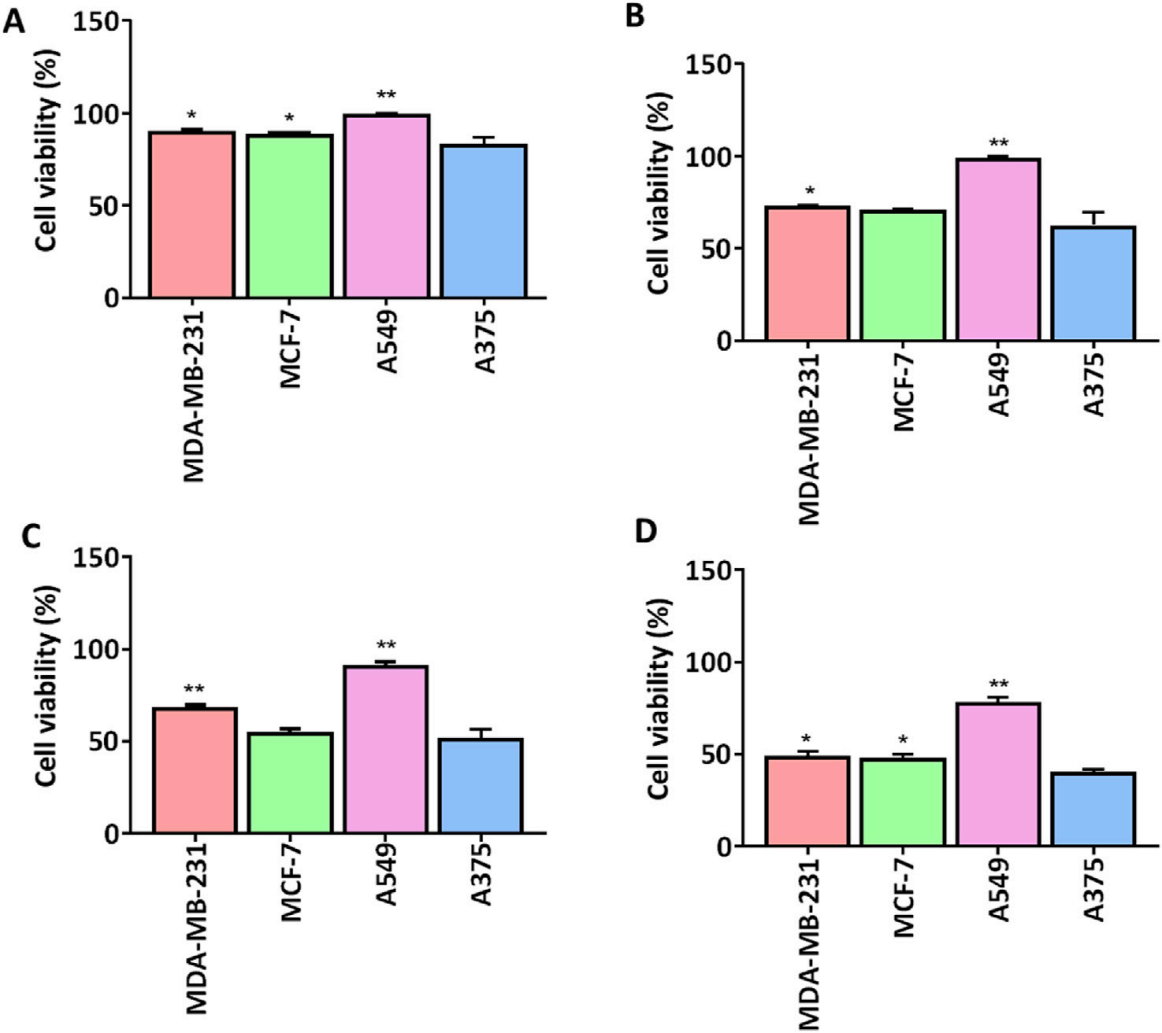
Comparison of cell viability (%) of **3g** at different concentrations in different human cell lines. **3g** was tested for cell viability (*n* = 3) at four different concentrations **(A)** 1 µM, **(B)** 5 µM, **(C)** 10 µM, **(D)** 20 µM. Cell viability graphs are expressed as mean ± SD (*n* = 3) and plotted using GraphPad^TM^ Prism 8.0 software. A375 cells were compared against other cancer cells for significance by applying One-way ANOVA with Dunnet’s multiple comparison test. *p < 0.05, **p < 0.001.

**FIGURE 3:**
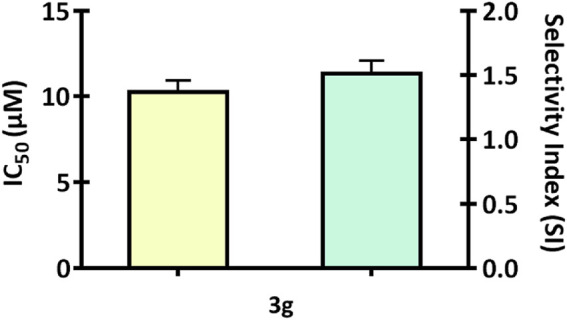
The bar graph illustrates the IC_50_ and Selectivity index (SI) of **3g** against A375, a human melanoma cell line. The graphs are expressed as mean ± SD (*n* = 3) and plotted using GraphPad™ Prism 8.0 software.

We screened a series of colchicine derivatives for their *in vitro* cytotoxicity in different cancer cell lines. We used SRB assay, a semi-automated colorimetric assay to evaluate the antiproliferative activity on different human cancer cell lines such as A549 (lung carcinoma), MCF-7 (breast carcinoma), MDAMB-231 (breast carcinoma) and A375 (melanoma) (Table 1). To access the cytotoxic activity levels, the effect of individual derivatives was assessed on a healthy non-cancerous cell line (HEK-293) to confirm their selectivity towards cancerous cells. Among all the derivatives, **3g** resulted in a marked reduction in cell viability with a comparatively improved selectivity index compared to colchicine. Detailed information about half-maximal inhibitory concentration, IC_50_ ± SD, and SI can be found in Table 1, demonstrating **3g** as a more selective cytotoxic agent against human melanoma cells than colchicine. It has improved selectivity (SI = 1.52) with IC_50_ (10.35 ± 0.56) (Table 1) and exhibits 60% growth inhibition at 20 µM in A375 cells (Figure 2). Additionally, **3g** exhibited potent cytotoxicity against MCF-7 cells with IC_50_ = 15.69 ± 0.39 and a selectivity index of 1.005 (Table 1). The IC_50_ and Selectivity index (SI) of **3g** against A375, a human melanoma cell line, as shown in Figure 3. The IC_50_ of Colchicine against mentioned human cancer cell lines has also been shown in Supplementary Table S1 along with Paclitaxel *via* MTT assay.

**TABLE 1:** Half-maximal inhibitory concentration (IC_50_, µmol/L) and selectivity index (SI) values of synthesized colchicine derivatives against various human cancer cell lines and a normal cell line using sulphorhodamine B assay.

	A375		MCF-7		MDA-MB 231		A549		HEK-293
Compounds	IC_50_ (µM)	SI	IC_50_ (µM)	SI	IC_50_ (µM)	SI	IC_50_ (µM)	SI	IC_50_ (µM)
Colchicine	0.076 ± 0.01	0.802	4.44 ± 0.28	0.013	4.83 ± 0.31	0.012	0.174 ± 0.028	0.35	0.061 ± 0.002
3a	>50		40.55 ± 0.91	0.73	>50		>50		29.66 ± 0.07
3b	>50		44.72 ± 0.64	0.85	>50		>50		38.33 ± 1.36
3c	>50		44.75 ± 0.68	0.79	>50		>50		35.61 ± 2.97
3d	>50		43.79 ± 0.33	0.82	>50		>50		36.09 ± 1.79
3e	>50		45.073 ± 0.73	0.89	>50		>50		40.29 ± 1.71
3f	28.27 ± 2.19	0.62	26.39 ± 2.79	0.67	46.59 ± 0.86	0.38	>50		17.81 ± 1.30
3g	10.35 ± 0.56	1.52	15.69 ± 0.39	1.005	24.92 ± 2.70	0.63	>50		15.78 ± 0.81
3h	35.16 ± 2.79	0.75	49.92 ± 2.72	0.53	>50		>50		26.55 ± 2.65
3i	>50		>50				>50		39.52 ± 1.36
3j	>50		>50		>50		>50		38.97 ± 0.79

The IC_50_ data are expressed as mean ± SD (*n* = 3) and were determined using GraphPad™. Prism 5.0 software. The selectivity index (SI) was calculated HEK-293 cell line (human embryonic kidney cells).

### 3.3 3g reduces colony-forming ability of A375 cells

**FIGURE 4:**
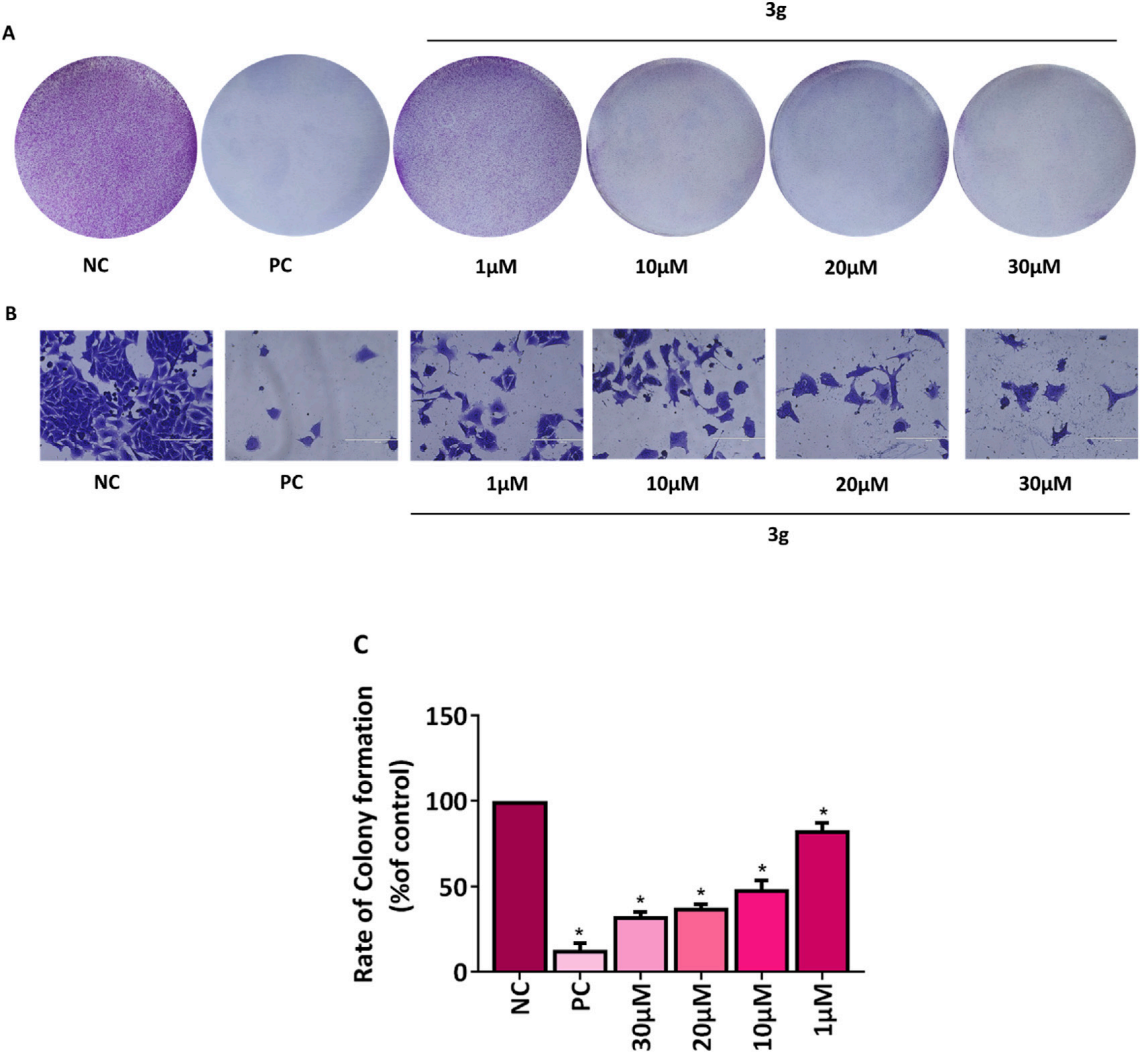
**3g** inhibits colony formation in A375 cells. **(A,B)** Representative images of colony growth inhibition of A375 cells treated with an indicated concentration of **3g** for 48 h. Camptothecin (0.1 µM) was used as a positive control (PC) (Rodríguez-Berna et al., 2014). **(A)** No magnification, **(B)** 20x, Sacle Bar 200 µm. **(C)** Colonies were quantified using ImageJ software (ver. 1.49 V), and graphs were plotted as mean ± SD (*n* = 3) using GraphPad™ Prism 8.0 software. The statistical significance was determined by comparing the treated samples with an untreated negative control (NC) using One-way ANOVA with Dunnet’s multiple comparison test. *p < 0.001 in comparison to NC.

The ability of individual cells to endure, multiply, and form colonies is determined by a colony formation assay used to assess how pharmaceuticals or radiation therapy affect the viability and proliferation of cells (Stone et al., 2016; Pouget et al., 2022). The antiproliferative effect of **3g** was examined using colony formation assay. The A375 cells were treated with various concentrations of **3g** (1 µM, 10 µM, 20 µM, 30 µM) for 48 h. It was observed that treatment with **3g** induced the irreversible growth arrest ability and significantly attenuated the size, number of colonies, and colony growth of A375 cells compared to untreated control cells (Figure 4). The percentage of colony growth formation was 100% in untreated control cells wheras it was 37.5% in case of **3g** at 20 µM in A375 cells.

### 3.4 3g reduces migration potential of A375 cells

**FIGURE 5:**
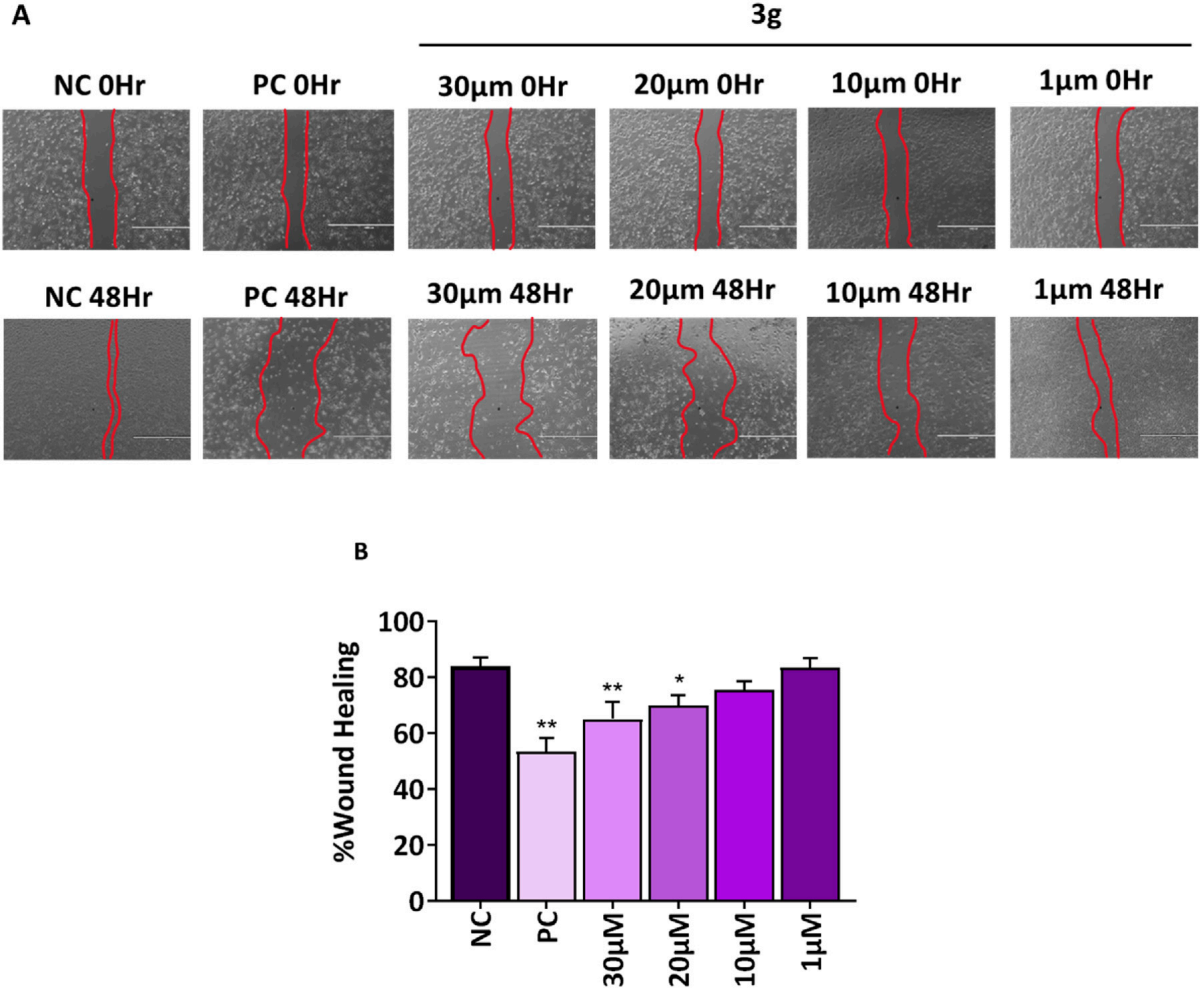
**3g** inhibits the migration of A375 cells into the wound region after 48h treatment with indicated concentrations. **(A)** Representative images from a bright field microscope (4x, Scale bar 1000 µm) showing inhibition of A375 cell invasion *in vitro* scratch/wound healing assay with indicated concentrations. Camptothecin (0.1 µM) was used as a positive control (PC). **(B)** The decrease in wound closure or gap filling with an increase in concentration of **3g** was quantified using ImageJ software (ver 1.49 V) and plotted as mean ± SD (*n* = 3) using GraphPad™ Prism 8.0 software. The statistical significance was determined by comparing the treated samples with an untreated negative control (NC) using One-way ANOVA with Dunnet’s multiple comparison test. *p < 0.05, **p < 0.001 in comparision to NC.

Tumor cell invasion and metastases are significantly influenced by cell migration (Shi et al., 2024). A fundamental characteristic of a chemotherapeutic agent is its ability to inhibit cell migration and invasion and its capacity to induce targeted cell death in cancerous tissues. Agents for anti-metastatic therapy may include molecules that block the migration of cancer cells (Anderson et al., 2019). Wound healing assay was performed to analyze the effect of compound **3g** on the wound closure rate and migration potential of cells. The A375 cells were treated with various concentrations of **3g** (30 µM, 20 μM, 10 µM, and 1 µM) for 48 h. **3g** was found to decrease the migration of A375 cells toward the margin of the wound, resulting in poor closure of the gap and healing of the wound compared to untreated control cells (Figure 5). The percentage of wound closure was 84% in untreated control cells whereas 69% in case of **3g** at 20 µM in A375 cells (Figure 5).

Colchicine blocks cell division by disrupting microtubules, and the spindle microtubules are more sensitive to colchicine than the interphase microtubules. It penetrates the cells and equilibrates with the external colchicine rapidly; however, a more extended period is required to attain saturation. Differential sensitivity at different stages of cell division as it binds to microtubules dissociates to tubulin dimers. For instance, colchicine at a concentration of 50 nM blocks almost all the prophase, metaphase, and anaphase cells at the mitosis cell cycle. This abnormal mitotic cycle is sometimes called C-mitosis or Colchicine-mitosis (Taylor, 1965). Moreover, it can inhibit the function of several ion channels and alter the membrane potential of the mitochondria, resulting in the release of proapoptotic factors like caspases, cytochrome-C, and apoptotic features, leading to cell cycle arrest and death (Volbracht et al., 2001). Microtubule targeting agents (MTA) are also named antimitotic agents that bind to the tubulin in the microtubules and prohibit the proliferation of the cells by inhibiting or destabilizing agents or agents that bind to the colchicine (Jordan and Wilson, 2004; Arnst, 2018; Mirzaei et al., 2020). Tubulin-targeting agents and microtubule-associated proteins as targets have gained much attention since vincristine was approved by the US FDA in 1963 in cancer treatment and anti-cancer drug development (Lu et al., 2012; Khwaja et al., 2021). Many colchicine-based derivatives have been synthesized and generated to develop novel and increasing pharmacological profiles to improve their efficacies, toxicity, and therapeutic importance (Zhang et al., 2015; Majcher et al., 2018; Pyta et al., 2022; Iqbal Lone et al., 2024).

The study found that a derivative of colchicine, identified as **3g**, demonstrated enhanced effectiveness against human melanoma cells. Specifically, **3g** exhibited a selectivity index approximately two times higher than colchicine. We sought to further evaluate the efficacy of **3g** against human melanoma in A375 cells using other *in vitro* assays such as colony formation assay and wound healing assay. We found that **3g** reduced the number and growth of colonies of these cells. The percentage of colony growth formation was 100% in untreated control cells wheras it was 37.5% in case of **3g** at 20 µM in A375 cells. Moreover, **3g** decreased the migration of A375 cells toward the margin of the wound, resulting in poor wound healing. The percentage of wound closure was 84% in untreated control cells whereas 69% in case of **3g** at 20 µM in A375 cells. These findings suggest we may have identified a more selective colchicine derivative targeting melanoma cells. This increased selectivity could reduce toxicity to healthy cells, thereby enhancing its therapeutic potential. With its higher selectivity and effectiveness, compound **3g** could present a substantial advantage over colchicine as a treatment for melanoma, potentially resulting in improved outcomes and reduced side effects.

### 3.5 Molecular docking

**FIGURE 6:**
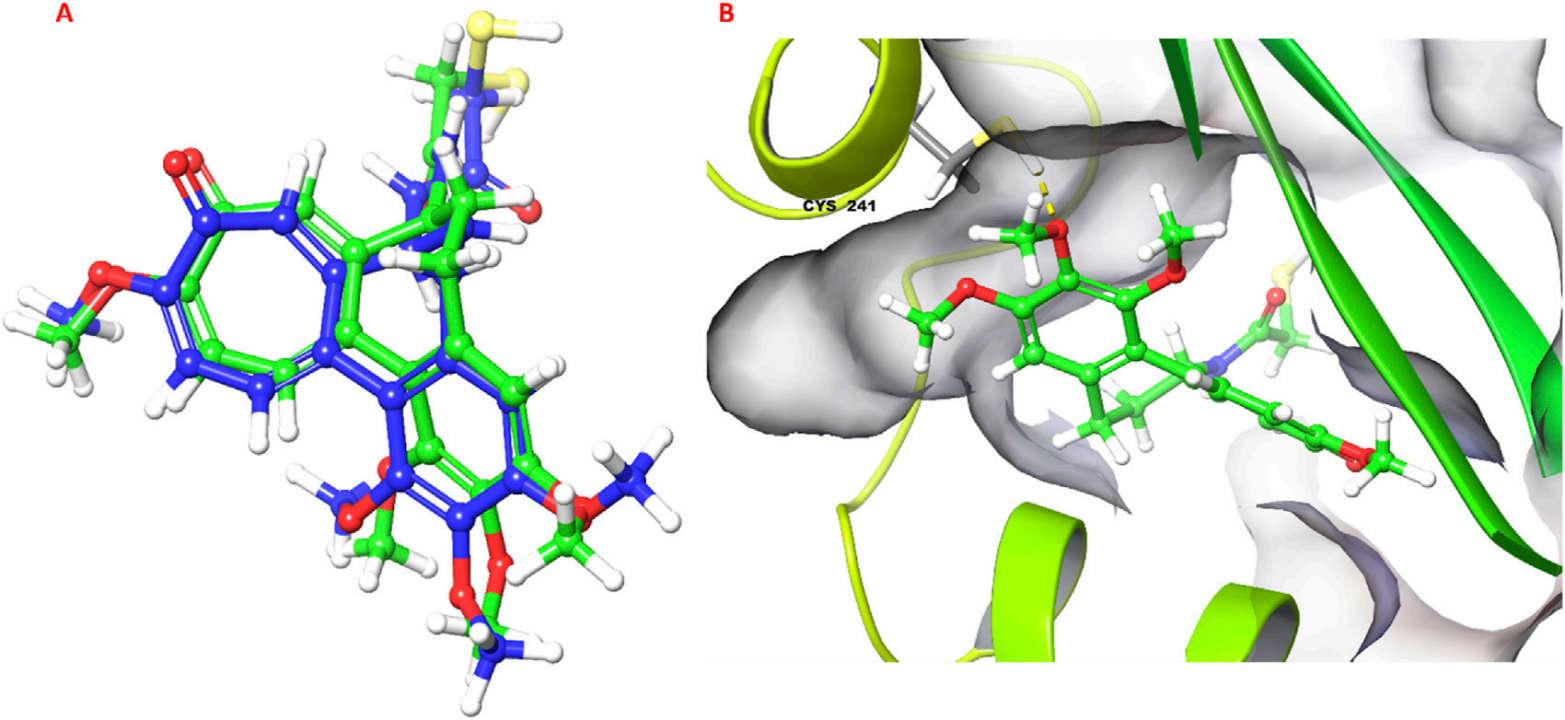
Validation of docking protocol. **(A)** Superimposed pose of colchicine co-crystalized with tubulin (PDB ID: 1SA0) with respect to molecular docking pose; **(B)** 3D pose of colchicine obtained from molecular docking, hydrogen bond is shown as yellow dashed line.

**FIGURE 7:**
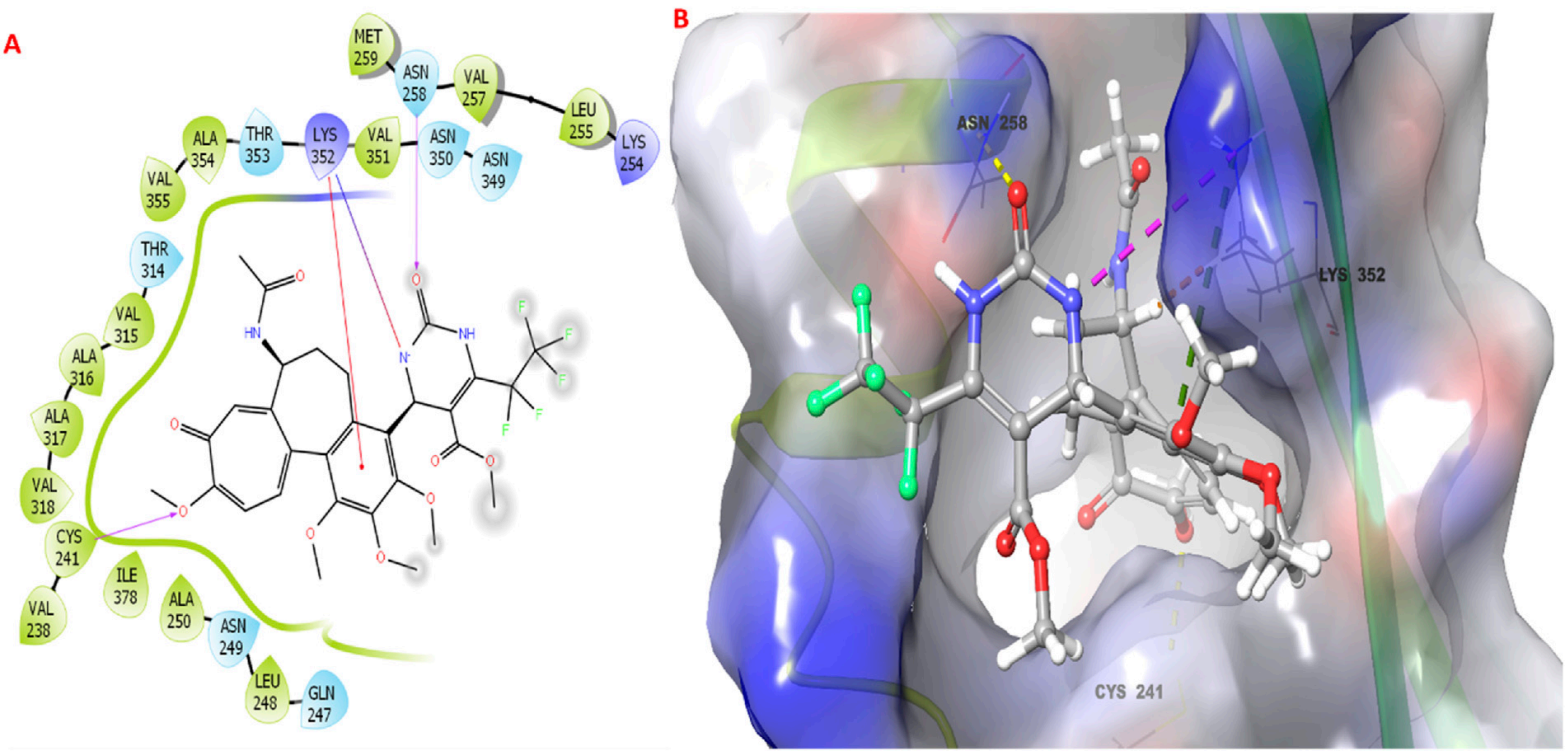
Molecular docking studies of colchicine derivative **3g** with the targeted protein. **(A,B)** 2D & 3D poses of colchicine derivative.

The molecular docking study investigated the binding interactions between colchicine, its derivative, and chain B of the tubulin protein. The docking protocol successfully reproduced the binding pose of the co-crystallized ligand with a low RMSD value of 1.33 Å, demonstrating the accuracy of the docking approach (Figure 6). Additionally, molecular docking of colchicine derivative (binding energy −6.498 kcal/mol) showed a comparable pose to colchicine (binding energy −7.963 kcal/mol). The colchicine derivative formed a hydrogen bond with Cys241, Asn258 and a salt bridge with Lys352 (Figure 7). Notably, the interaction with Cys241 is also shown by co-crystalized colchicine in PDB ID 1SA0 (Figure 7). Therefore, these interactions might be important for binding the colchicine derivative with tubulin.

### 3.6 Pharmacokinetic properties of compound 3g

The pkCSM results provide a comprehensive overview of the pharmacokinetic and toxicity properties of the compound (Table 2). In terms of absorption, the compound exhibits moderate intestinal absorption in humans (75.376%) but has low water solubility (−5.195) and poor Caco2 permeability (−0.229). The compound is a substrate for P-glycoprotein and acts as an inhibitor for both P-glycoprotein I and II, which may influence its absorption and efflux. The distribution volume (VDss) is low (−1.367), and the fraction unbound in plasma is 0.148, indicating significant protein binding. The compound has poor blood-brain barrier (BBB) permeability (−2.15) and very low CNS permeability (−3.483), suggesting it is unlikely to cross into the central nervous system.

**TABLE 2:** Pharmacokinetic properties of compound **3g** predicted by pkCSM tool.

S. No.	Property	Model name	Predicted value	Unit
1.	Absorption	Water solubility	−5.195	Numeric (log mol/L)
2.	Absorption	Caco2 permeability	−0.229	Numeric (log Papp in 10^−6^ cm/s)
3.	Absorption	Intestinal absorption (human)	75.376	Numeric (% Absorbed)
4.	Absorption	Skin Permeability	−2.768	Numeric (log Kp)
5.	Absorption	P-glycoprotein substrate	Yes	Categorical (Yes/No)
6.	Absorption	P-glycoprotein I inhibitor	Yes	Categorical (Yes/No)
7.	Absorption	P-glycoprotein II inhibitor	Yes	Categorical (Yes/No)
8.	Distribution	VDss (human)	−1.367	Numeric (log L/kg)
9	Distribution	Fraction unbound (human)	0.148	Numeric (Fu)
10.	Distribution	BBB permeability	−2.15	Numeric (log BB)
11.	Distribution	CNS permeability	−3.483	Numeric (log PS)
12.	Metabolism	CYP2D6 substrate	No	Categorical (Yes/No)
13.	Metabolism	CYP3A4 substrate	Yes	Categorical (Yes/No)
14.	Metabolism	CYP1A2 inhibitior	No	Categorical (Yes/No)
15.	Metabolism	CYP2C19 inhibitior	No	Categorical (Yes/No)
16.	Metabolism	CYP2C9 inhibitior	No	Categorical (Yes/No)
17.	Metabolism	CYP2D6 inhibitior	No	Categorical (Yes/No)
18.	Metabolism	CYP3A4 inhibitior	Yes	Categorical (Yes/No)
19.	Excretion	Total Clearance	−0.214	Numeric (log mL/min/kg)
20.	Excretion	Renal OCT2 substrate	No	Categorical (Yes/No)
21.	Toxicity	AMES toxicity	No	Categorical (Yes/No)
22.	Toxicity	Max. tolerated dose (human)	−0.139	Numeric (log mg/kg/day)
23.	Toxicity	hERG I inhibitor	No	Categorical (Yes/No)
24.	Toxicity	hERG II inhibitor	Yes	Categorical (Yes/No)
25.	Toxicity	Oral Rat Acute Toxicity (LD50)	2.358	Numeric (mol/kg)
26.	Toxicity	Oral Rat Chronic Toxicity (LOAEL)	1.285	Numeric (log mg/kg_bw/day)
27.	Toxicity	Hepatotoxicity	No	Categorical (Yes/No)
28.	Toxicity	Skin Sensitisation	No	Categorical (Yes/No)
29.	Toxicity	*T.Pyriformis* toxicity	0.291	Numeric (log ug/L)
30.	Toxicity	Minnow toxicity	1.622	Numeric (log mM)

Regarding metabolism, the compound is a substrate for CYP3A4 but not CYP2D6. It inhibits CYP3A4 but does not inhibit other major CYP enzymes, which may reduce the risk of drug-drug interactions. Excretion data indicate low total clearance (−0.214), and the compound is not a substrate for renal OCT2, suggesting renal excretion may not be a major elimination pathway. Toxicity profiling shows the compound is non-toxic in AMES tests and non-hepatotoxic. It does not inhibit hERG I but inhibits hERG II. The compound has moderate acute toxicity (LD_50_ = 2.358) and low chronic toxicity (LOAEL = 1.285) in rats. It is non-sensitizing to the skin and shows low toxicity in T. Pyriformis (0.291) and moderate toxicity in minnows (1.622). PredMS predicts the compound to be metabolically stable in human liver micosomes.

## 4 Conclusion

In summary, a series of novel colchicine analogs derivatized at the C-4 position was utilized to obtain *via* multi-component reaction methods and screen for *in vitro* cytotoxicity against human cancer cell lines and normal human embryonic kidney cells (HEK-293) to evaluate for their anti-cancer potency. The MCR method has high utility in drug discovery, especially for structurally optimizing complex natural products for their biological properties (Kaur et al., 2017). Derivative **3g** reduced cell viability and showed a significantly improved selectivity index against melanoma compared to colchicine. It has a selectivity index of 1.52 with 60% growth inhibition at 20 µM in melanoma cancer cells with IC_50_ of 10.35 ± 0.56. Additionally, **3g** exhibited significant cytotoxicity against MCF-7 cells with IC_50_ of 15.69 ± 0.39 and a selectivity index of 1.005. Among the tested derivatives, compound **3g** is the most promising candidate, as elucidated by the molecular docking studies, demonstrating significant potential as a colchicine-based anti-cancer agent that can overcome drug resistance and improve efficacy and pharmacokinetic properties for cancer therapy treatment strategies.

## Data Availability

The original contributions presented in the study are included in the article/Supplementary Material, further inquiries can be directed to the corresponding authors.
