# Supplementary material for: Design, synthesis and antiproliferative activity of novel colchicine derivatives: selective inhibition of melanoma cell proliferation
Source: Front Pharmacol. 2025 Jun 27;16:1528235. doi: 10.3389/fphar.2025.1528235 (PMC12246756; doi:10.3389/fphar.2025.1528235)
Supplement: Supplementary file 1 [file DataSheet1.pdf]

Supporting information

Design, synthesis and antiproliferative activity of novel colchicine derivatives: selective inhibition of melanoma cell proliferation

Puneet Kumar† [1,3], Tusharika Kotra† [2,3], Waseem I. Lone [1,3], Yassir Arfath [2,3], Harshita Tiwari[4], Ashutosh Kumar Shukla[4], Zabeer Ahmed[2,3] Sheikh Rayees[2,3,*], Jasha Momo H. Anal [1,3,*]

[1]Natural Products and Medicinal Chemistry Division, CSIR-Indian Institute of Integrative Medicine, Jammu-180001, India.

[2]Pharmacology Division, CSIR-Indian Institute of Integrative Medicine, Jammu-180001, India.

[3]Academy of Scientific and Innovative Research (AcSIR), Ghaziabad-201002, India.

[4]Drug Chemistry Research Centre, Kanadia Road, Indore, 452005, India

Table of Content

**S.No**	**Figure**
1	HPLC chromatogram of Colchicine **(1)**
2	Mass of Colchicine-aldehyde **(2)**
3	^{1}H NMR of Colchicine-aldehyde **(2)**
4	^{13}C NMR of Colchicine-aldehyde (**2**)
5	Mass of **(3a)**
6	^{1}H NMR of **(3a)**
7	^{13}C NMR of **(3a)**
8	Mass of **(3b)**
9	^{1}H NMR of **(3b)**
10	^{13}C NMR of **(3b)**
11	Mass of **(3c)**
12	^{1}H NMR of **(3c)**
13	^{13}C NMR of **(3c)**
14	Mass of **(3d)**
15	^{1}H NMR of **(3d)**
16	^{13}C NMR of **(3d)**
17	Mass of **(3e)**
18	^{1}H NMR of **(3e)**
19	^{13}C NMR of **(3e)**
20	Mass of **(3f)**
21	^{1}H NMR of **(3f)**

22	^{13}C NMR of **(3f)**
23	Mass of **(3g)**
24	^{1}H NMR of **(3g)**
25	^{13}C NMR of **(3g)**
26	Mass of **(3h)**
27	^{1}H NMR of **(3h)**
28	^{13}C NMR of **(3h)**
29	Mass of **(3i)**
30	^{1}H NMR of **(3i)**
31	^{13}C NMR of **(3i)**
32	Mass of **(3j)**
33	^{1}H NMR of **(3j)**
34	^{13}C NMR of **(3j)**
35	MTT Results

HPLC Data of Colchicine

SHIMADZU LabSolutions

Analysis Report

<Sample Information>

Sample Name	: COL-P		
Sample ID	: COL-P		
Data Filename	: 26-06-2024_02.lcd		
Method Filename	: 19-06-2023_Gradiant-02.lcm		
Batch Filename	: 26.06..2024-01.lcb		
Vial #	: 1-5	Sample Type	: Unknown
Injection Volume	: 5 uL		
Date Acquired	: 26/06/2024 10:32:43	Acquired by	: Dr. Nagaraju Nekkala
Date Processed	: 26/06/2024 14:58:28	Processed by	: Dr. Nagaraju Nekkala

<Chromatogram>

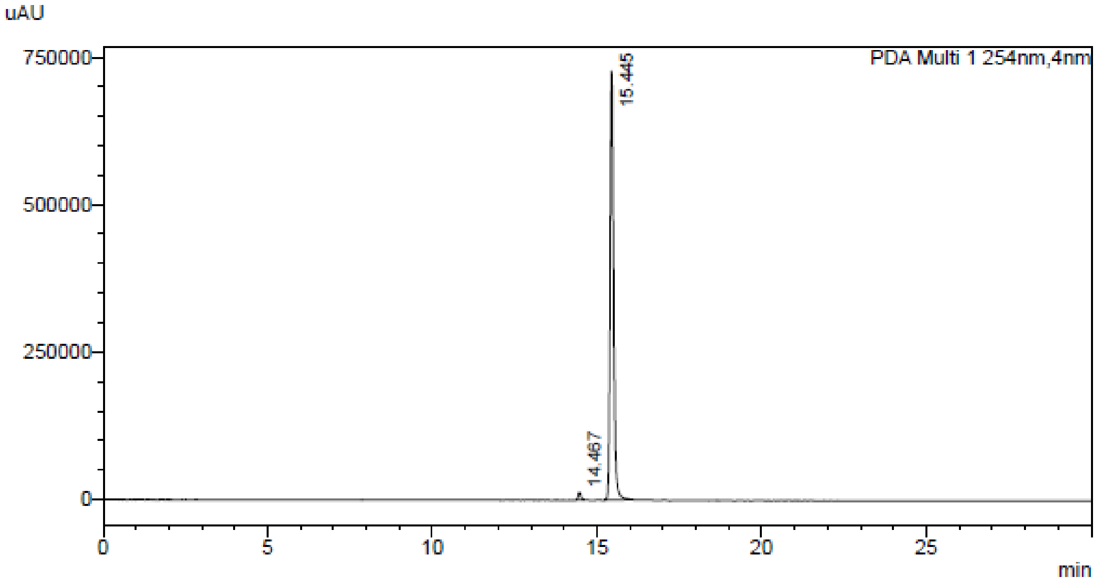

<Peak Table>

PDA Ch1 254nm

Peak#	Ret. Time	Area	Area%
1	14.467	70660	1.296
2	15.445	5380129	98.704
Total		5450789	100.000

HRMS of Colchicine-aldehyde (2)

Elemental Composition Report **Page 1**

Single Mass Analysis
Tolerance = 100.0 PPM / DBE: min = -1.5, max = 50.0
Element prediction: Off
Number of isotope peaks used for i-FIT = 3

Monoisotopic Mass, Even Electron Ions
20 formula(e) evaluated with 1 results within limits (up to 3 closest results for each mass)
Elements Used:
C: 0-23 H: 0-100 N: 0-1 O: 0-7

COL-CHO
QMI DIVISION, CSIR-IIIM JAMMU
Xevo G2-XS QTOF YFC2015
28-May-2024
14:59:18
280524_06 4 (0.104)
1: TOF MS ES+
1.58e+007

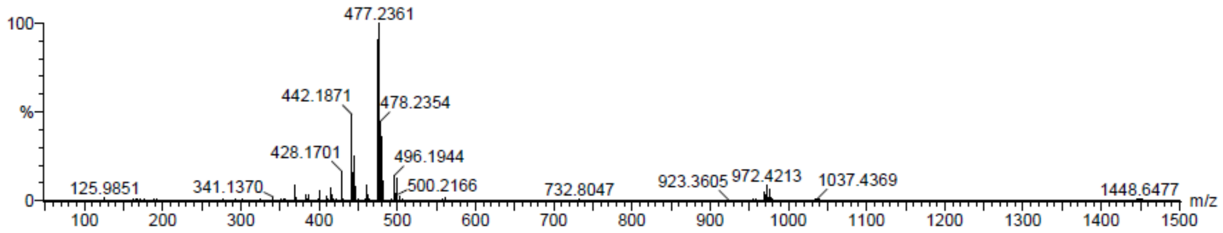

Minimum:				-1.5				
Maximum:		2.0	100.0	50.0				
Mass	Calc. Mass	mDa	PPM	DBE	i-FIT	Norm	Conf(%)	Formula
428.1701	428.1709	-0.8	-1.9	11.5	1029.8	n/a	n/a	C23 H26 N O7

^{1}H NMR of Colchicine-aldehyde (2)

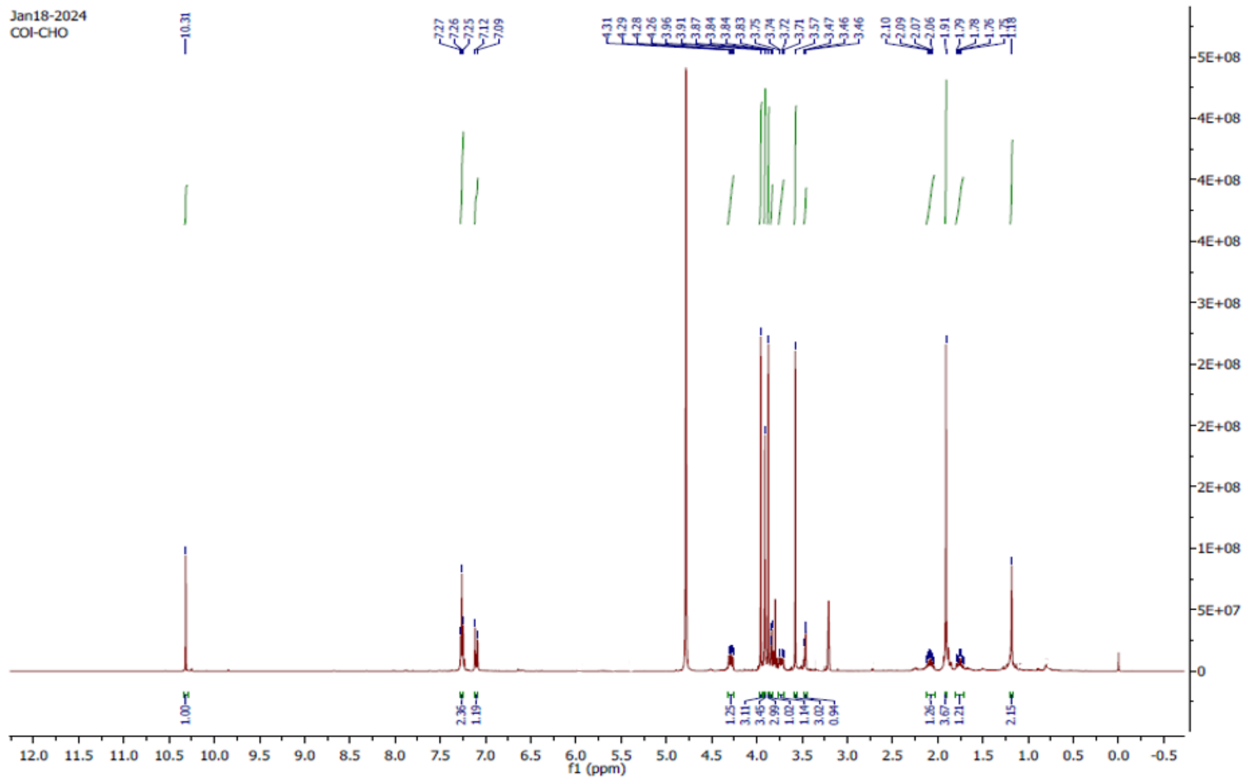

^{13}C NMR of Colchicine-aldehyde (2)

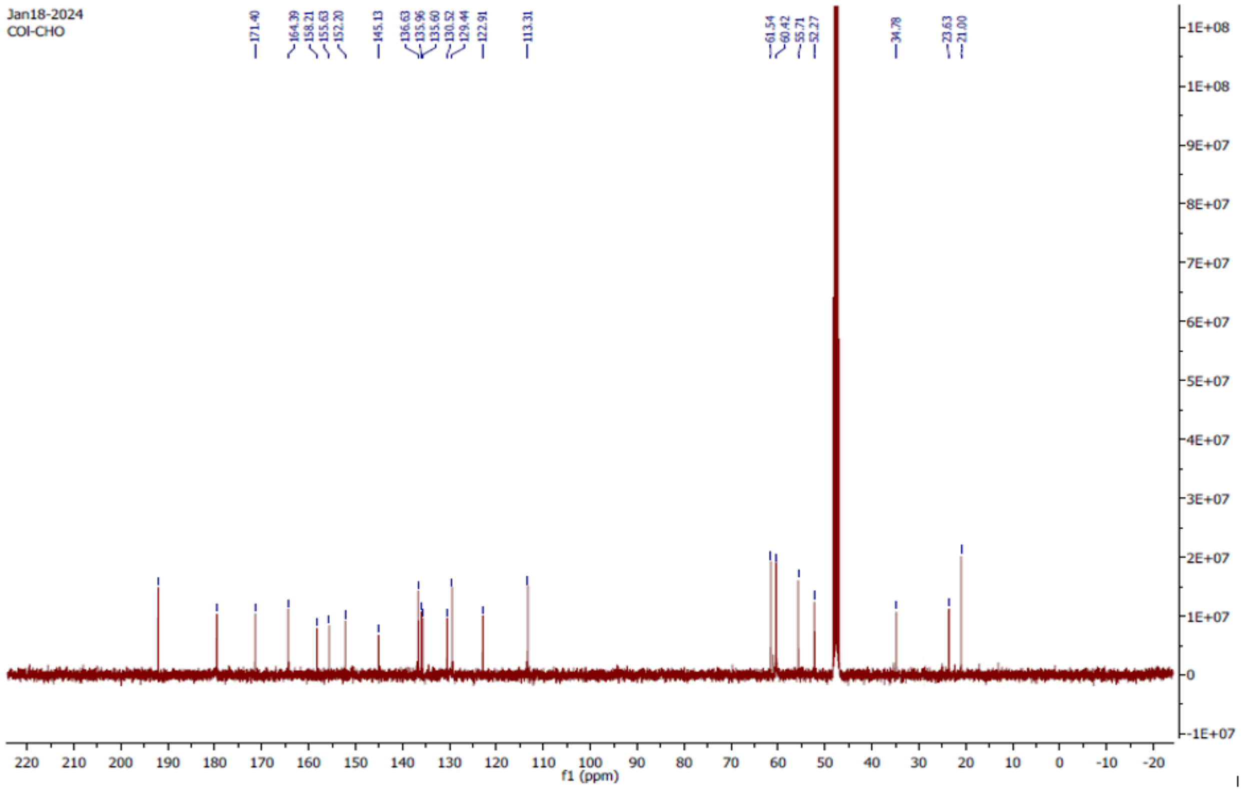

HRMS of 3a

Elemental Composition Report **Page 1**

Single Mass Analysis
Tolerance = 100.0 PPM / DBE: min = -1.5, max = 50.0
Element prediction: Off
Number of isotope peaks used for i-FIT = 3

Monoisotopic Mass, Even Electron Ions
76 formula(e) evaluated with 1 results within limits (up to 3 closest results for each mass)
Elements Used:
C: 0-30 H: 0-100 N: 0-3 O: 0-8 S: 0-1

DP-02 QMI DIVISION, CSIR-IIIM JAMMU 28-May-2024
Xevo G2-XS QTOF YFC2015 15:12:25
280524_11 6 (0.138) 1: TOF MS ES+
1.01e+008

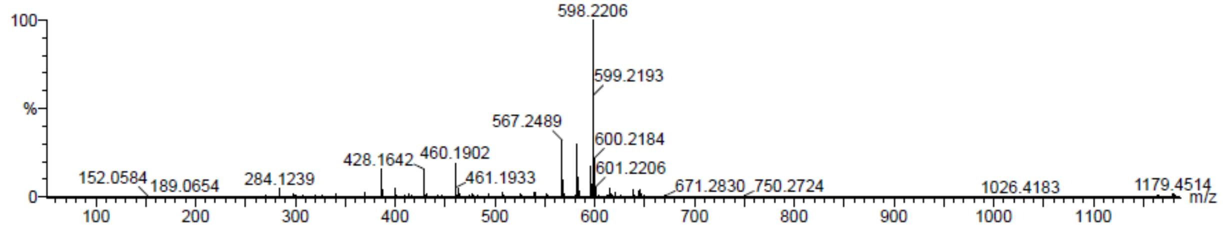

Minimum:				-1.5				
Maximum:		2.0	100.0	50.0				
Mass	Calc. Mass	mDa	PPM	DBE	i-FIT	Norm	Conf(%)	Formula
598.2206	598.2223	-1.7	-2.8	14.5	866.2	n/a	n/a	C30 H36 N3 O8 S

^{1}H NMR of 3a

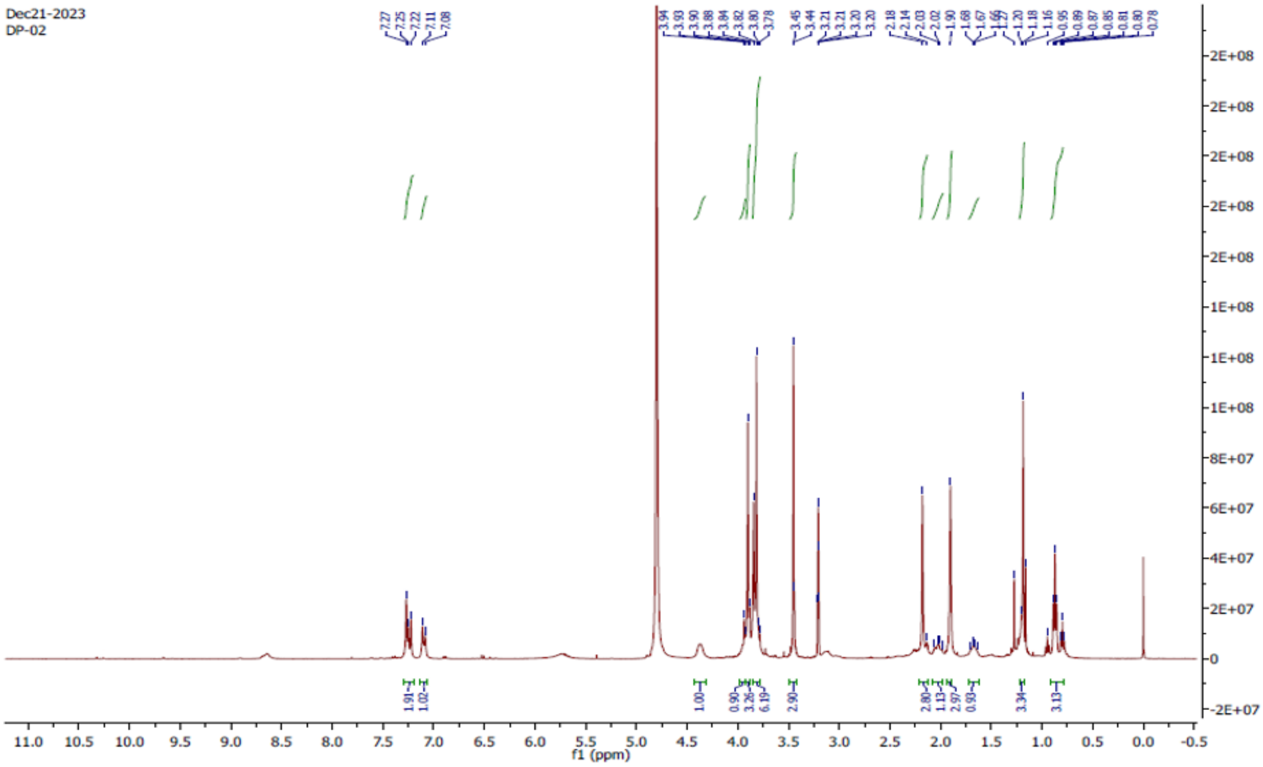

^{13}C NMR of 3a

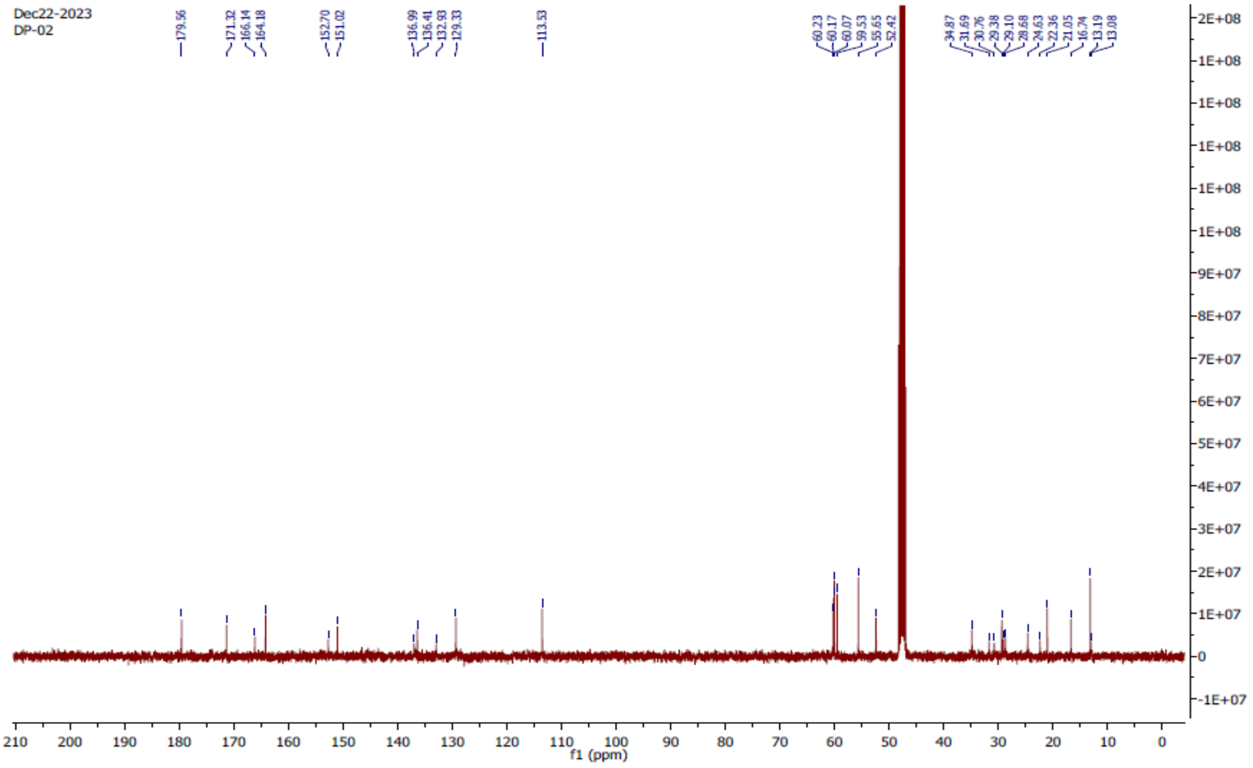

Mass of 3b

Sample Information

**Sample Name**	**: DP-03**	**Sample ID**	**: DP-03**
**Tray#**	**: 1**	**Vial#**	**: 10**
**Injection Volume**	**: 2**	**Data File**	**: 22-05-2024 -JSM_03.lcd**
**Method File**	**: MASS SCANNN 13APRIL2021.lcm**	**Processed by**	**: System Administrator**
**Date Processed**	**: 5/22/2024 12:53:23 PM**		

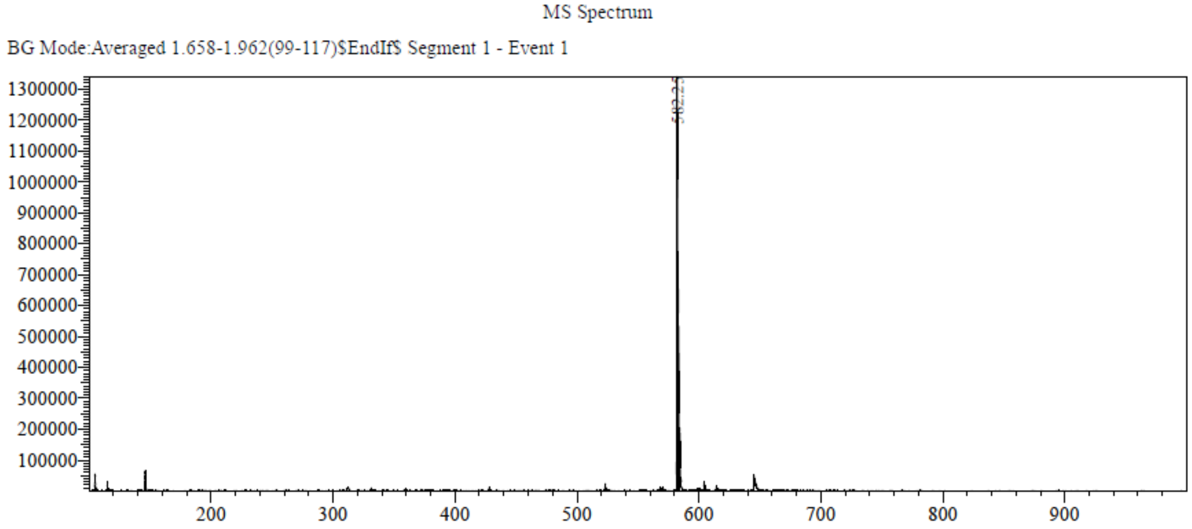

^{1}H NMR of 3b

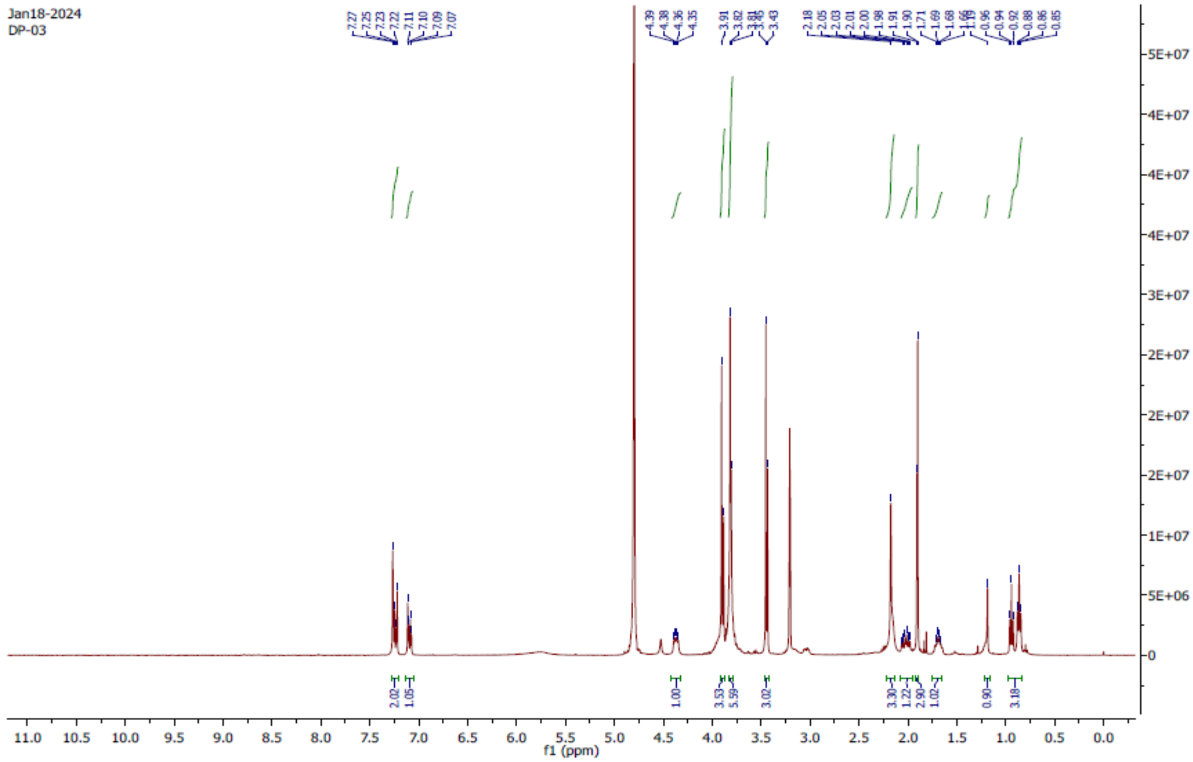

^{13}C NMR of 3b

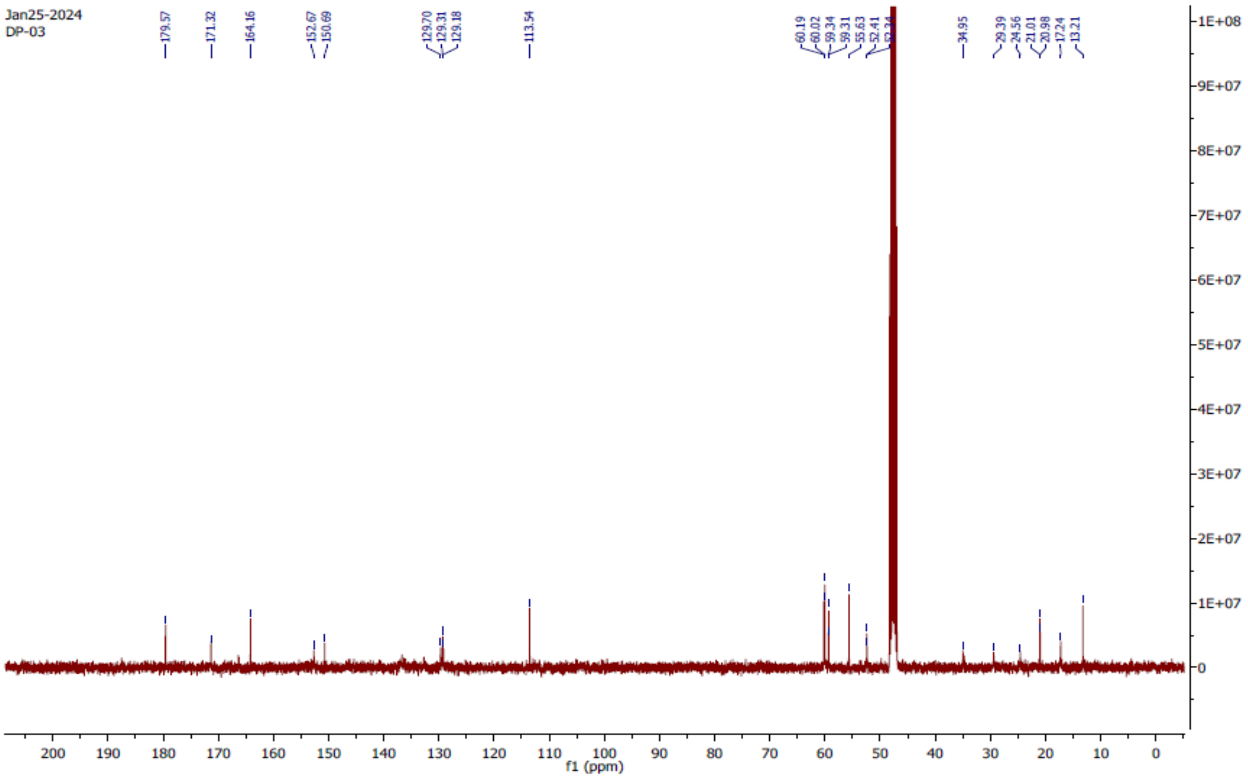
Jan25-2024
DP-03
179.57
171.32
164.16
152.67
150.69
129.70
129.31
129.18
113.54
60.19
60.02
59.34
59.31
55.63
52.41
34.95
29.39
24.56
21.01
20.98
17.24
13.21
f1 (ppm)

Mass of 3b

Sample Information

**Sample Name**	**: DP-05**	**Sample ID**	**: DP-05**
**Tray#**	**: 1**	**Vial#**	**: 11**
**Injection Volume**	**: 2**	**Data File**	**: 22-05-2024 -JSM_04.lcd**
**Method File**	**: MASS SCANNN 13APRIL2021.lcm**	**Processed by**	**: System Administrator**
**Date Processed**	**: 5/22/2024 12:53:36 PM**		

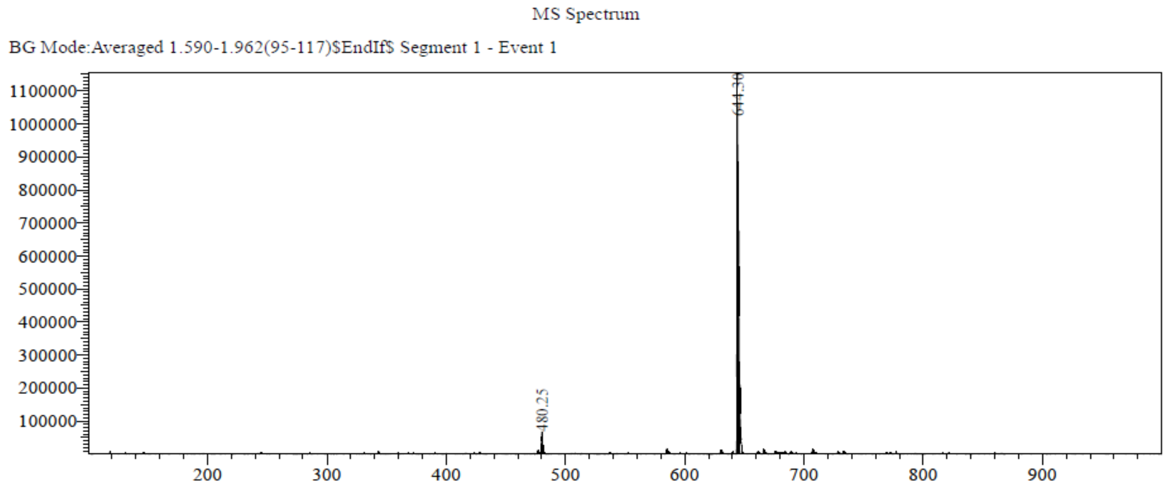

^{1}H NMR of DP-05

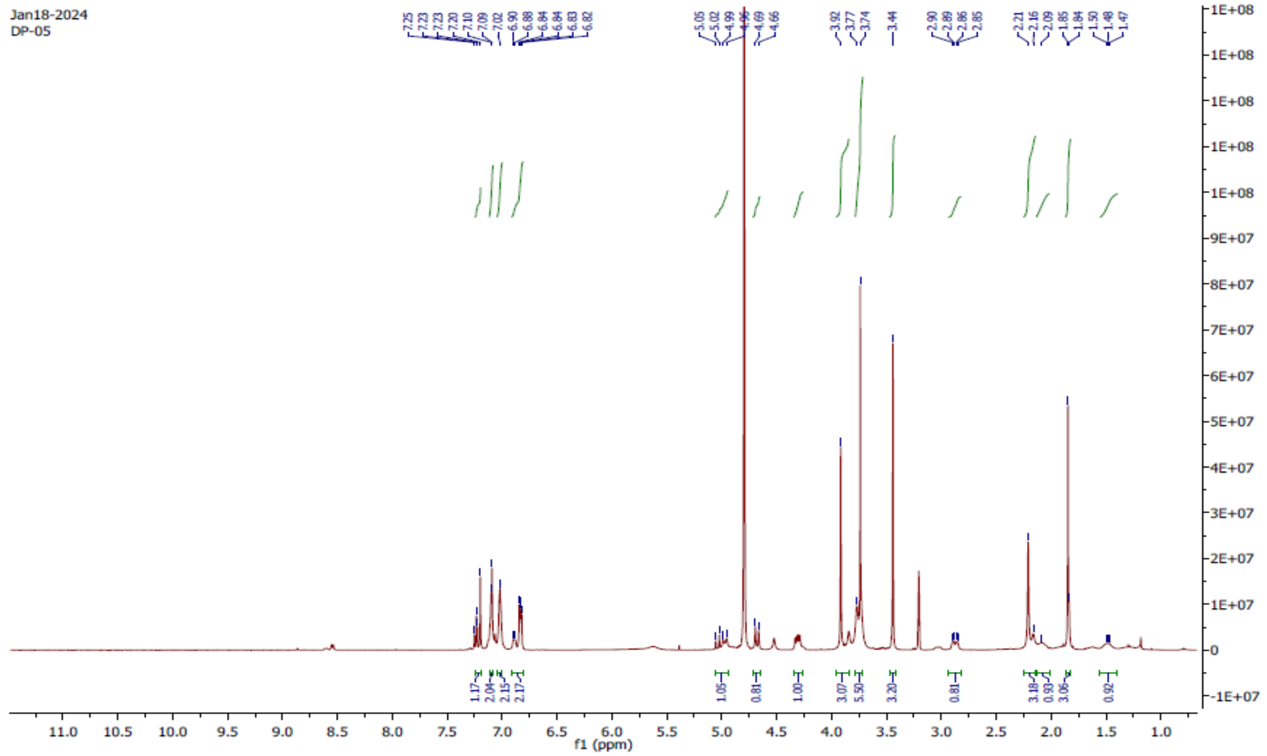

^{13}C NMR of 3b

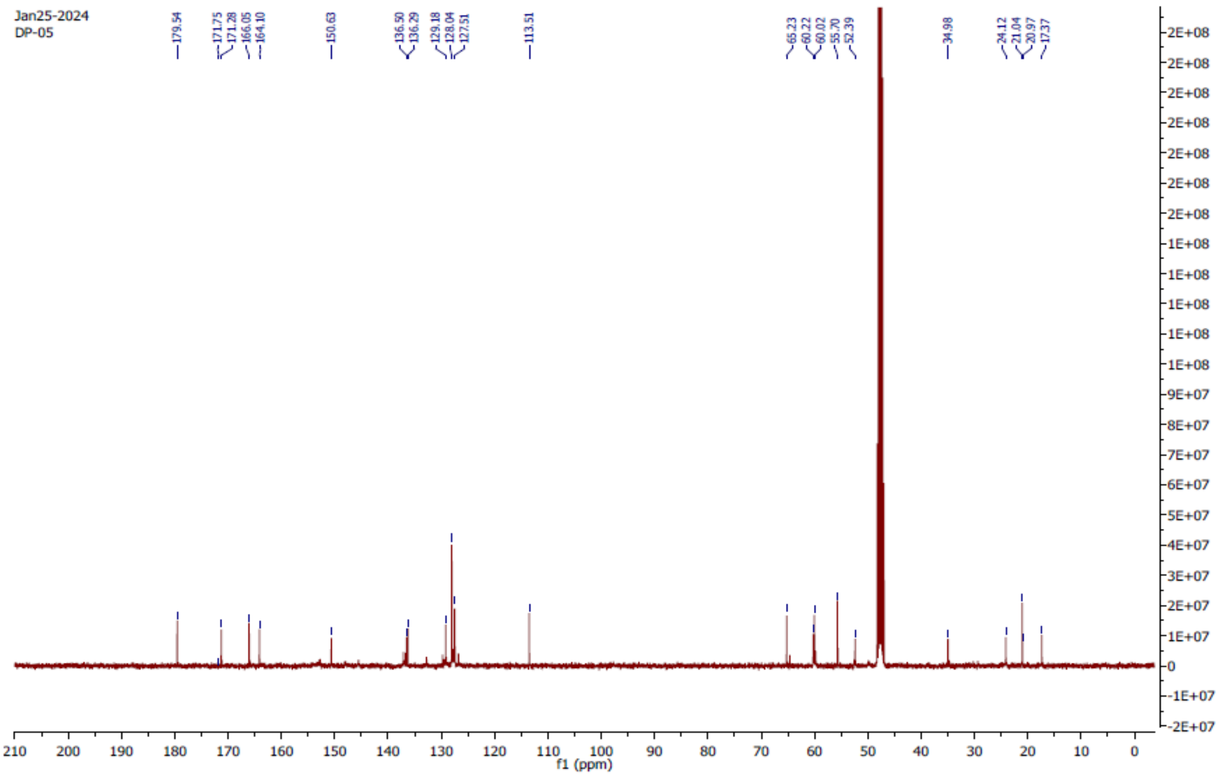

Jan25-2024
DP-05
179.54
171.75
171.28
166.05
164.10
150.63
136.50
136.29
129.18
128.04
127.51
113.51
65.23
60.22
60.02
55.70
52.39
34.98
24.12
21.04
20.97
17.37
f1 (ppm)

Mass of 3d

Sample Information

**Sample Name**	**: DP-06**	**Sample ID**	**: DP-06**
**Tray#**	**: 1**	**Vial#**	**: 12**
**Injection Volume**	**: 2**	**Data File**	**: 22-05-2024 -JSM_05.lcd**
**Method File**	**: MASS SCANNN 13APRIL2021.lcm**	**Processed by**	**: System Administrator**
**Date Processed**	**: 5/22/2024 12:53:49 PM**		

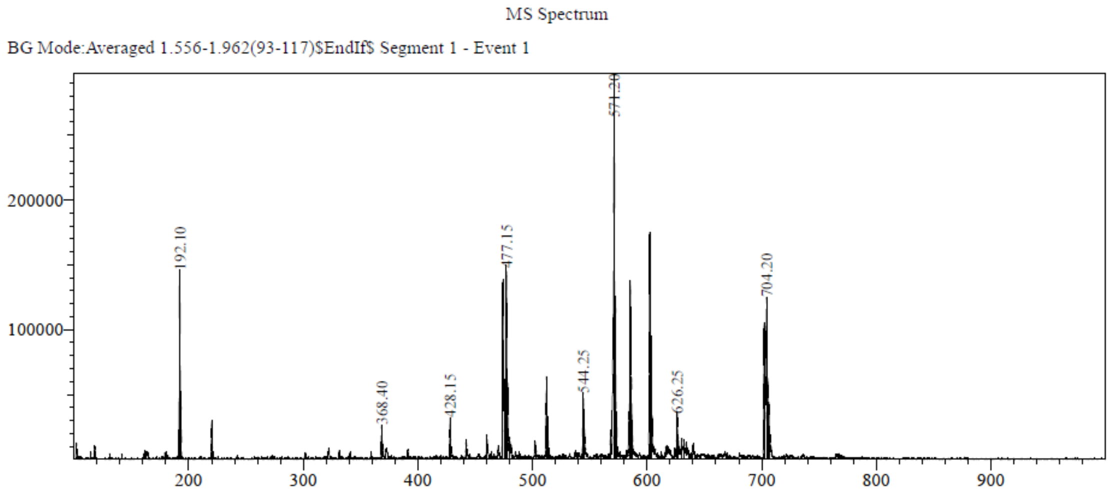

^{1}H NMR of 3d

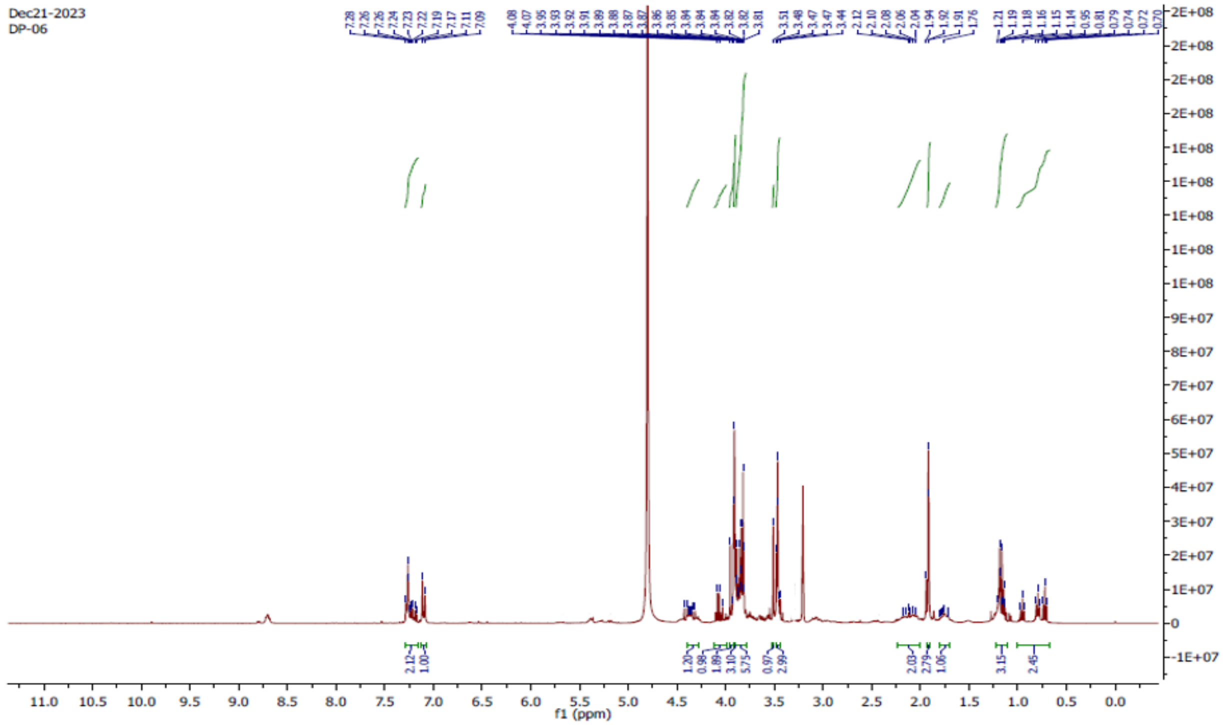

^{13}C NMR of 3d

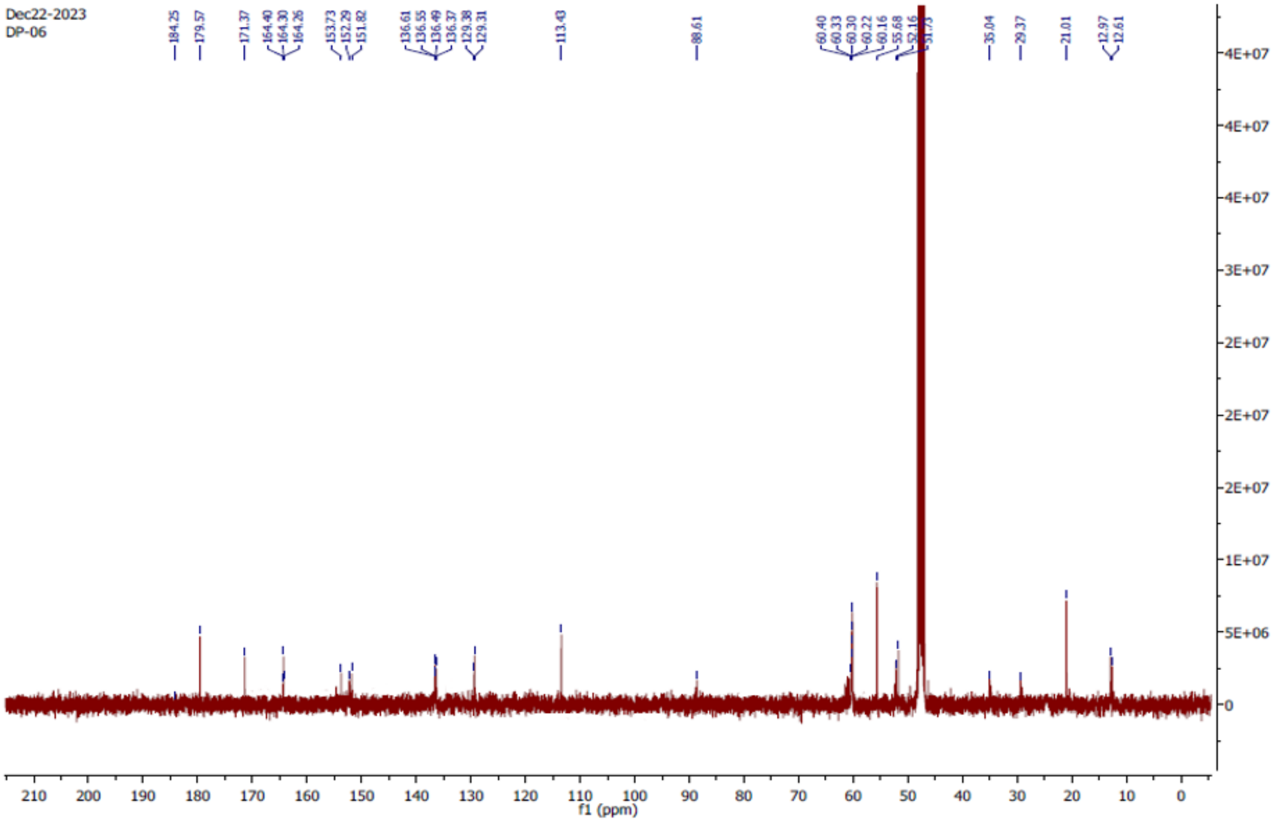
Dec22-2023
DP-06
184.25
179.57
171.37
164.40
164.30
164.26
153.73
152.29
151.82
136.61
136.55
136.49
136.37
129.38
129.31
113.43
88.61
60.40
60.33
60.30
60.22
60.16
55.68
52.16
51.73
35.04
29.37
21.01
12.97
12.61
f1 (ppm)

Mass of 3e

Elemental Composition Report

Single Mass Analysis

Tolerance = 100.0 PPM / DBE: min = -1.5, max = 50.0
Element prediction: Off
Number of isotope peaks used for i-FIT = 3

Monoisotopic Mass, Even Electron Ions
313 formula(e) evaluated with 1 results within limits (up to 3 closest results for each mass)
Elements Used:
C: 0-34 H: 0-100 N: 0-4 O: 0-9 Cl: 0-2 F: 0-1

DP-07
QMI DIVISION, CSIR-IIIM JAMMU
Xevo G2-XS QTOF YFC2015
28-May-2024
15:15:07
280524_12 6 (0.138)
1: TOF MS ES+
4.99e+007

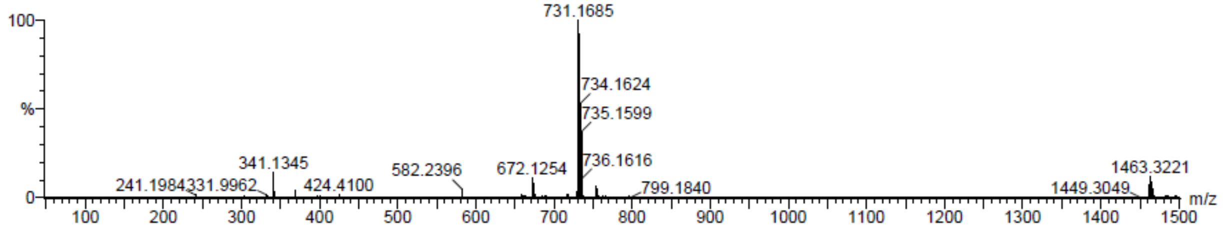

Minimum:				-1.5				
Maximum:		2.0	100.0	50.0				
Mass	Calc. Mass	mDa	PPM	DBE	i-FIT	Norm	Conf(%)	Formula
731.1685	731.1687	-0.2	-0.3	18.5	685.4	n/a	n/a	C34 H34 N4 O9 Cl2 F

^{1}H NMR of 3e

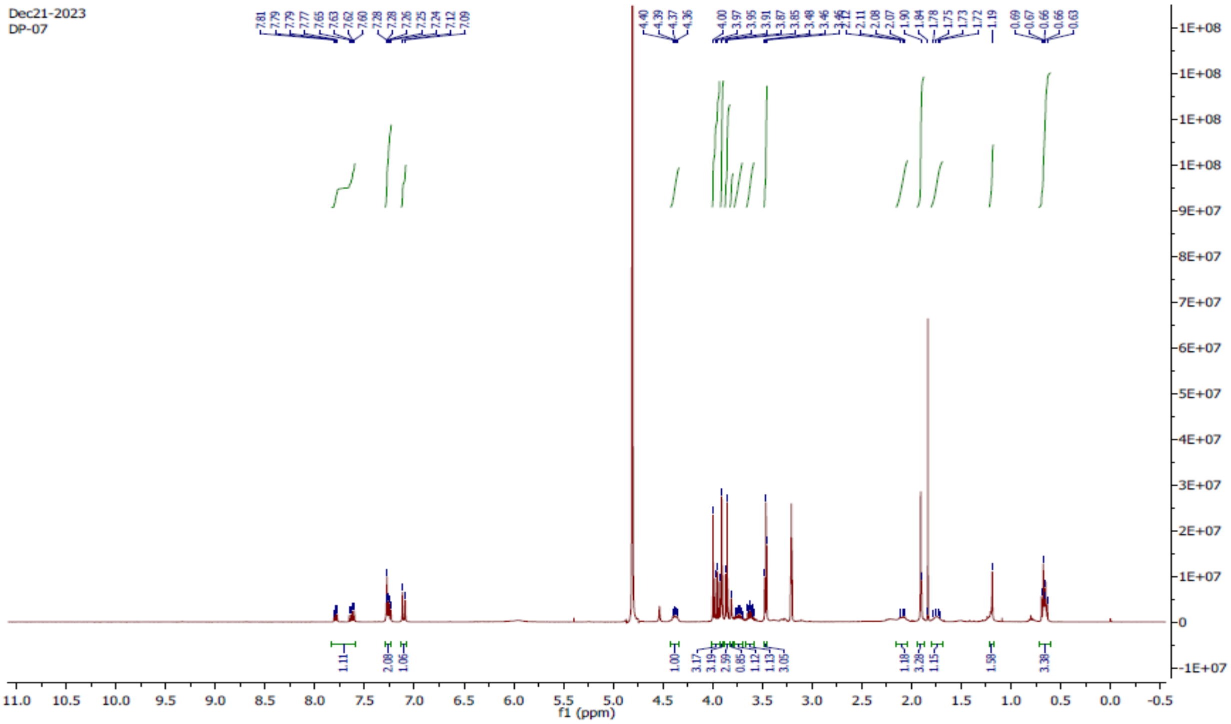

^{13}C NMR of 3e

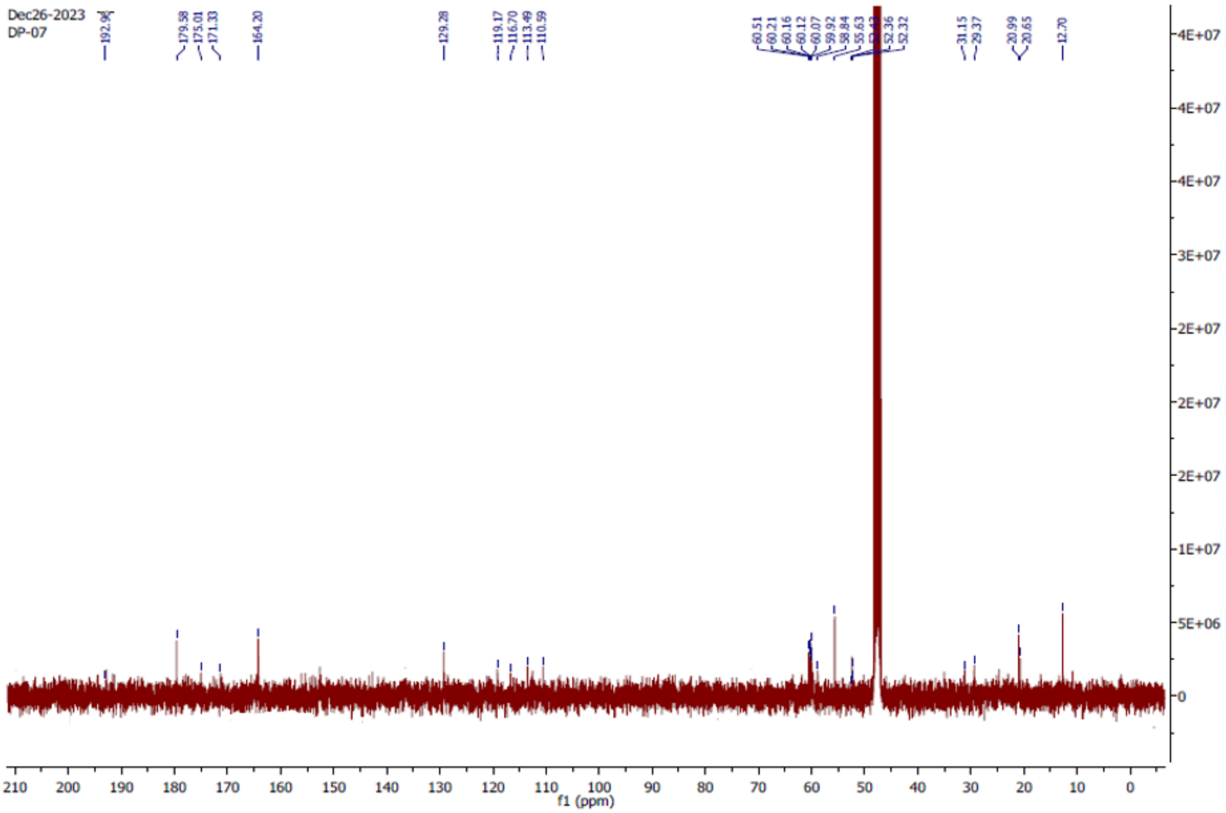
Dec26-2023
DP-07
192.9
179.58
175.01
171.33
164.20
129.28
119.17
116.70
113.49
110.59
60.51
60.21
60.16
60.12
60.07
59.92
58.84
55.63
52.43
52.36
52.32
31.15
29.37
20.99
20.65
12.70
4E+07
4E+07
4E+07
3E+07
2E+07
2E+07
2E+07
1E+07
5E+06
0
210 200 190 180 170 160 150 140 130 120 110 100 90 80 70 60 50 40 30 20 10 0
f1 (ppm)

Mass of 3f

Sample Information

**Sample Name**	**: DP-9A**	**Sample ID**	**: DP-9A**
**Tray#**	**: 1**	**Vial#**	**: 14**
**Injection Volume**	**: 2**	**Data File**	**: 22-05-2024 -JSM_07.lcd**
**Method File**	**: MASS SCANNN 13APRIL2021.lcm**	**Processed by**	**: System Administrator**
**Date Processed**	**: 5/22/2024 12:42:02 PM**		

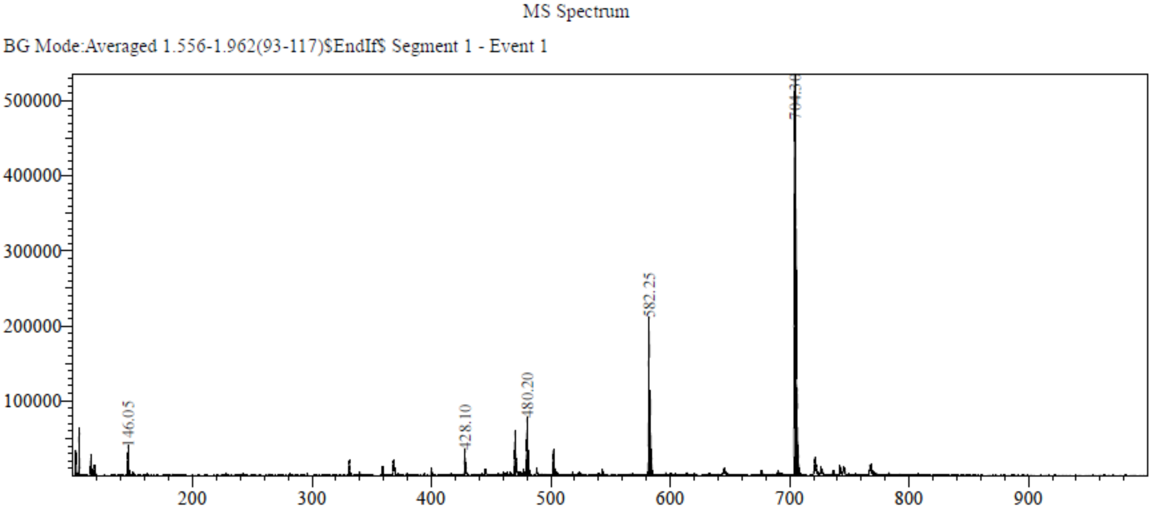

^{1}H NMR of 3f

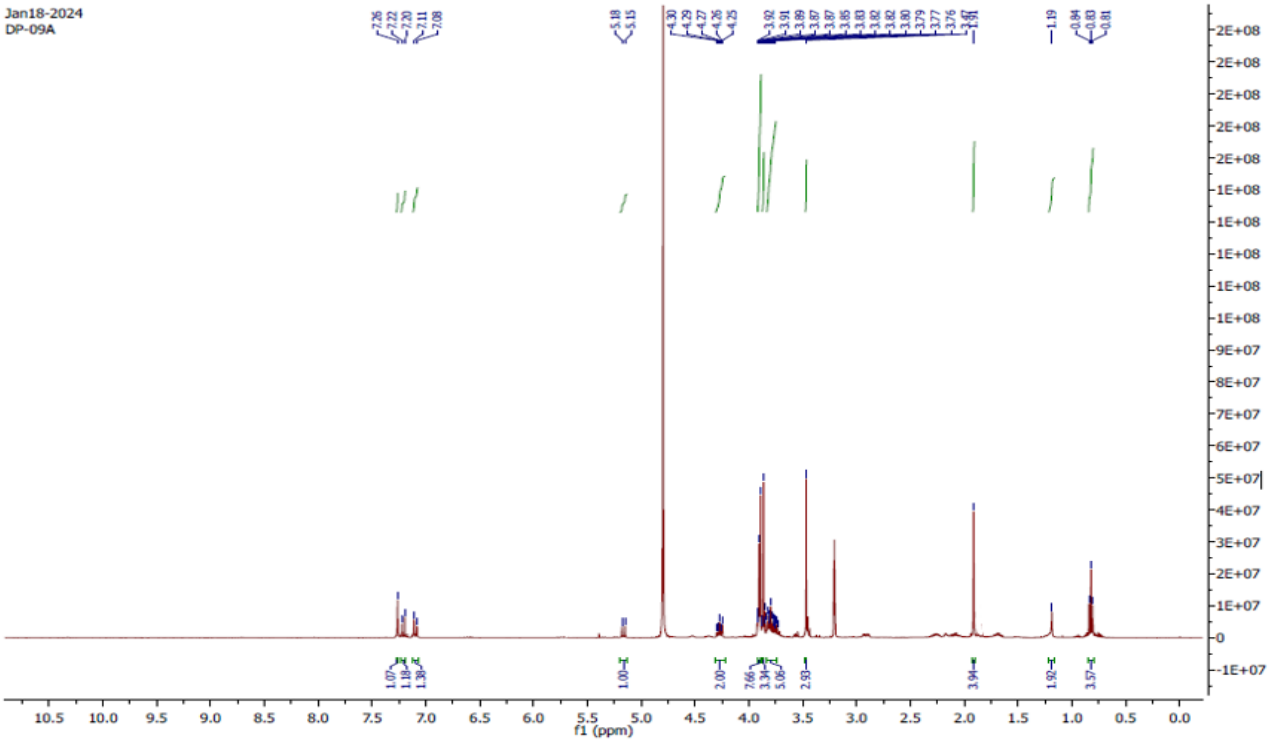

^{13}C NMR of 3f

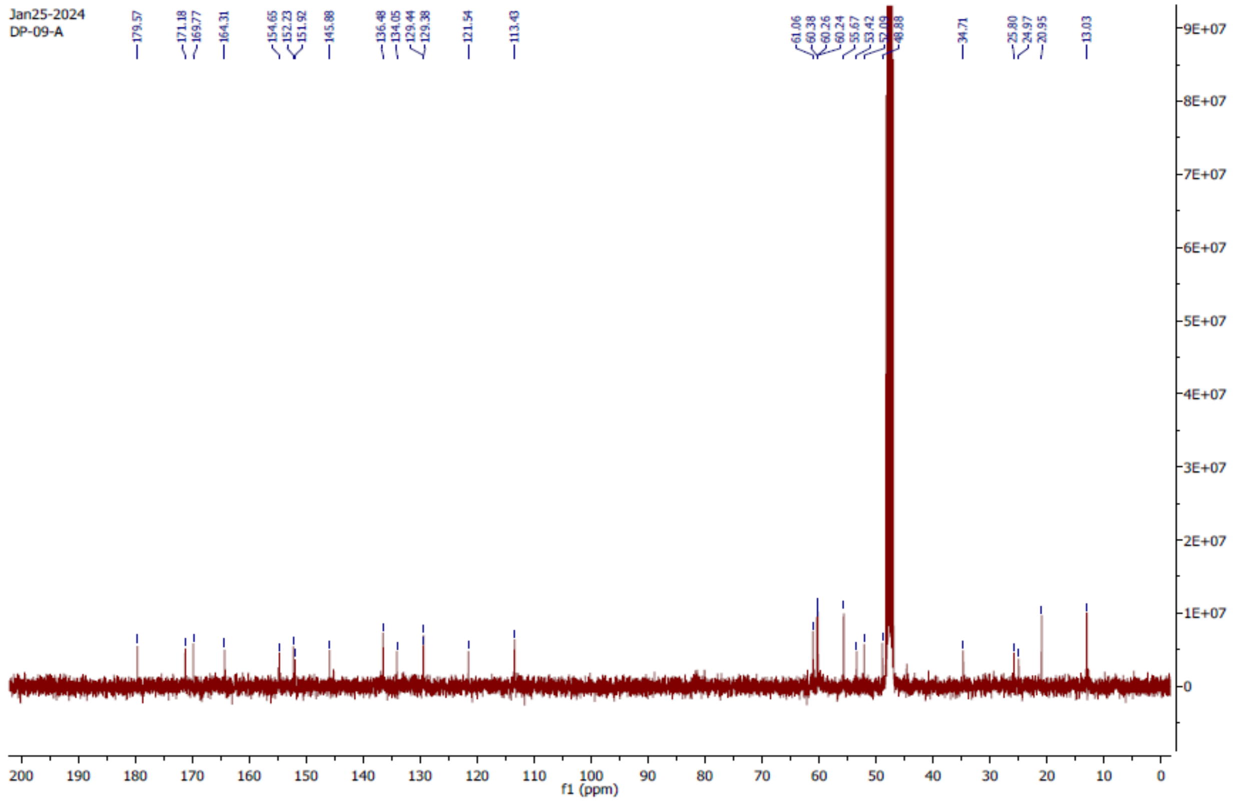

Jan25-2024
DP-09-A
179.57
171.18
169.77
164.31
154.65
152.23
151.92
145.88
136.48
134.05
129.44
129.38
121.54
113.43
61.06
60.38
60.26
60.24
55.67
53.42
52.09
48.88
34.71
25.80
24.97
20.95
13.03
9E+07
8E+07
7E+07
6E+07
5E+07
4E+07
3E+07
2E+07
1E+07
0
200 190 180 170 160 150 140 130 120 110 100 90 80 70 60 50 40 30 20 10 0
f1 (ppm)

Mass of 3g

**Sample Information**			
**Sample Name**	**: DP-9B**	**Sample ID**	**: DP-9B**
**Tray#**	**: 1**	**Vial#**	**: 15**
**Injection Volume**	**: 2**	**Data File**	**: 22-05-2024 -JSM_08.lcd**
**Method File**	**: MASS SCANNN 13APRIL2021.lcm**	**Processed by**	**: System Administrator**
**Date Processed**	**: 5/22/2024 12:45:35 PM**		

MS Spectrum

BG Mode:Averaged 1.590-1.962(95-117)$EndIf$ Segment 1 - Event 1

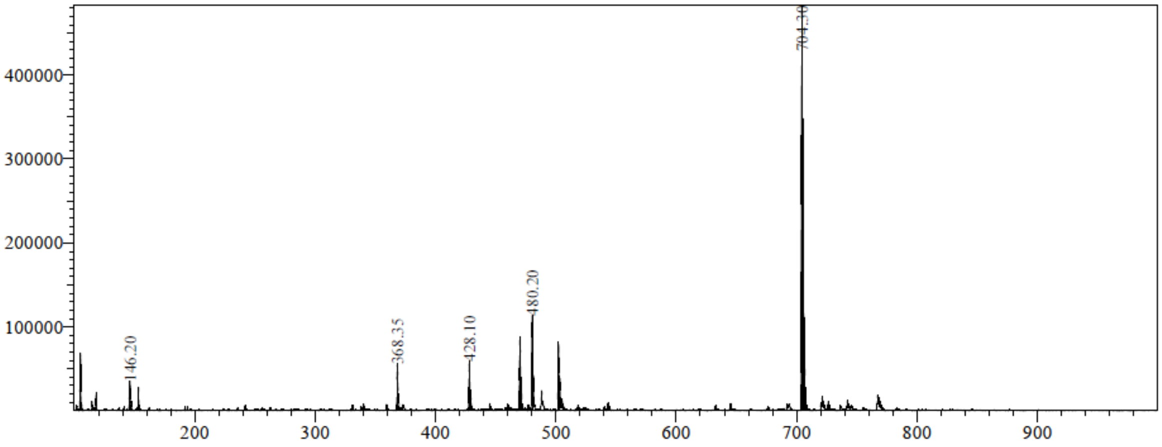

^{1}H NMR of 3g

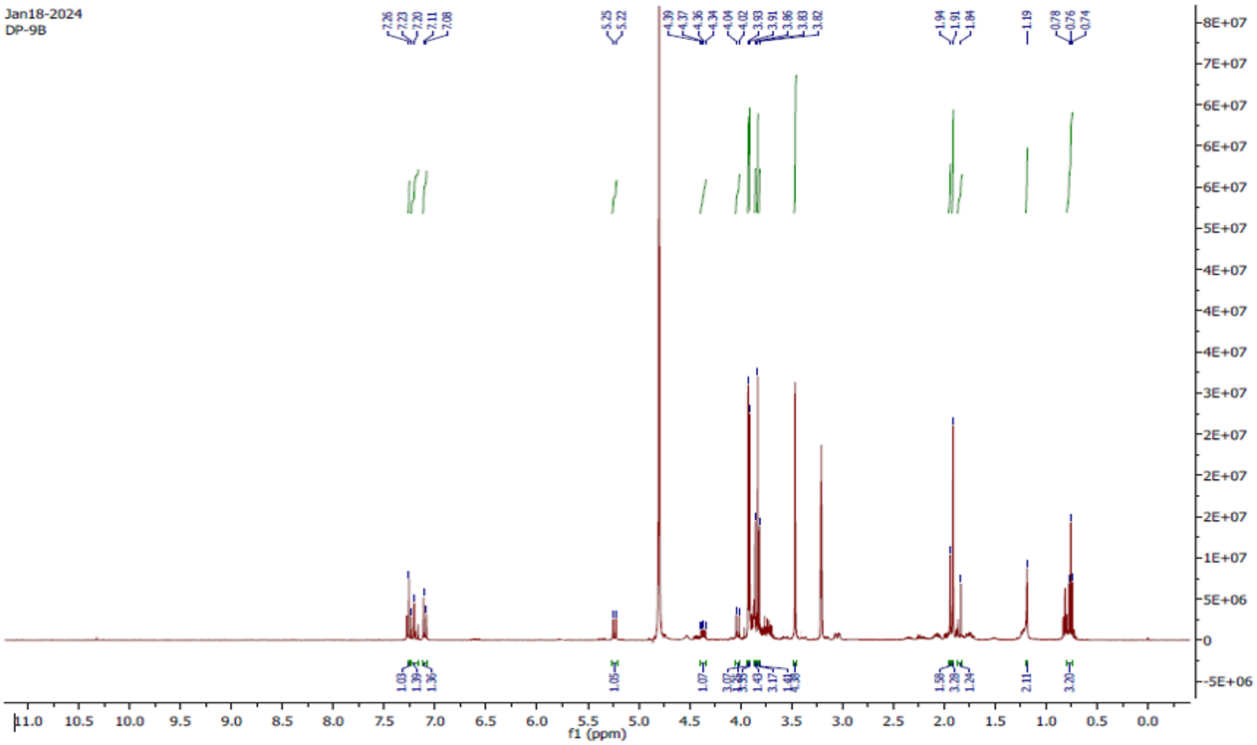

^{13}C NMR of 3g

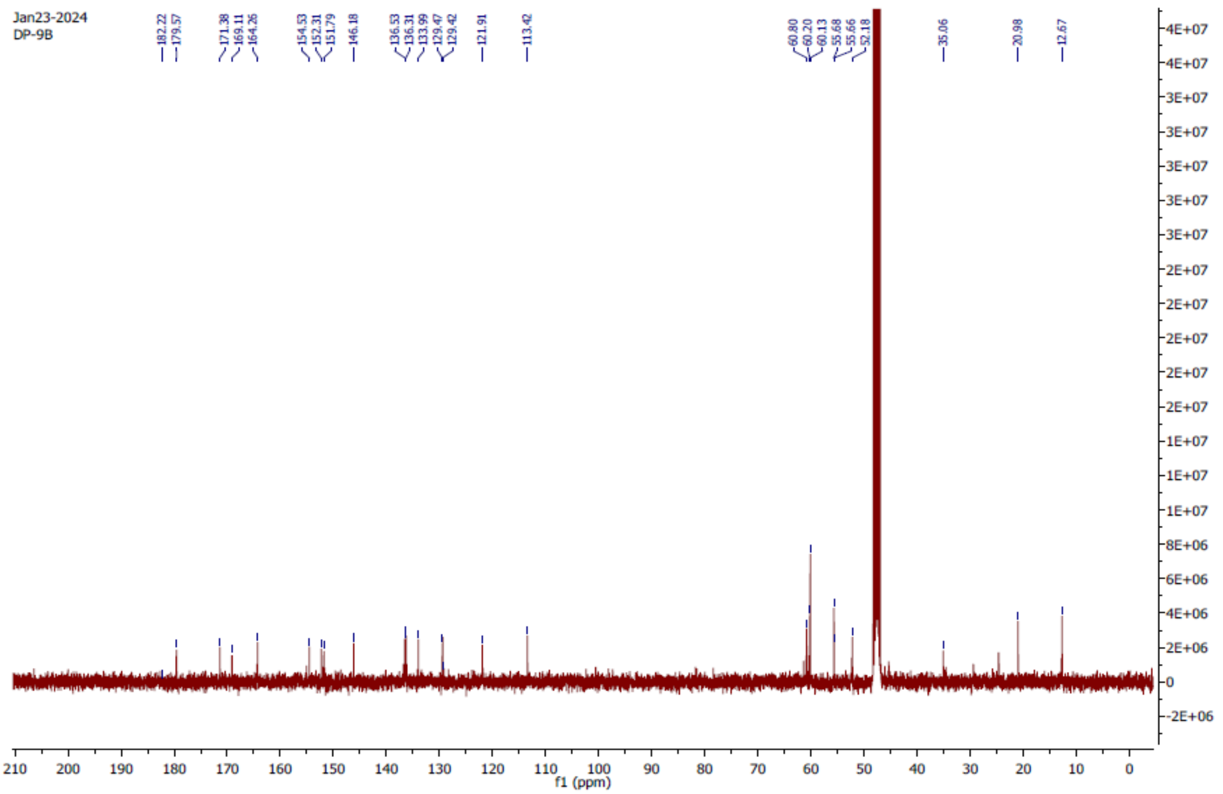

Jan23-2024
DP-9B
182.22
179.57
171.38
169.11
164.26
154.53
152.31
151.79
146.18
136.53
136.31
133.99
129.47
129.42
121.91
113.42
60.80
60.20
60.13
55.68
55.66
52.18
35.06
20.98
12.67
f1 (ppm)

Mass of 3h

**Sample Information**			
**Sample Name**	**: DP-10A**	**Sample ID**	**: DP-10A**
**Tray#**	**: 1**	**Vial#**	**: 16**
**Injection Volume**	**: 2**	**Data File**	**: 22-05-2024 -JSM_09.lcd**
**Method File**	**: MASS SCANNN 13APRIL2021.lcm**	**Processed by**	**: System Administrator**
**Date Processed**	**: 5/22/2024 1:00:11 PM**		

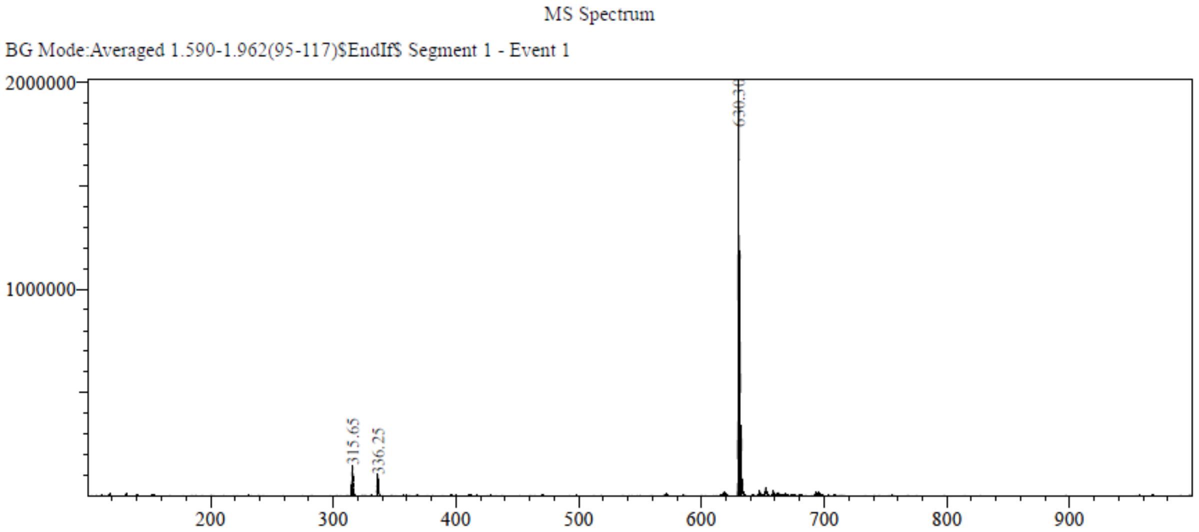

^{1}H NMR of 3h

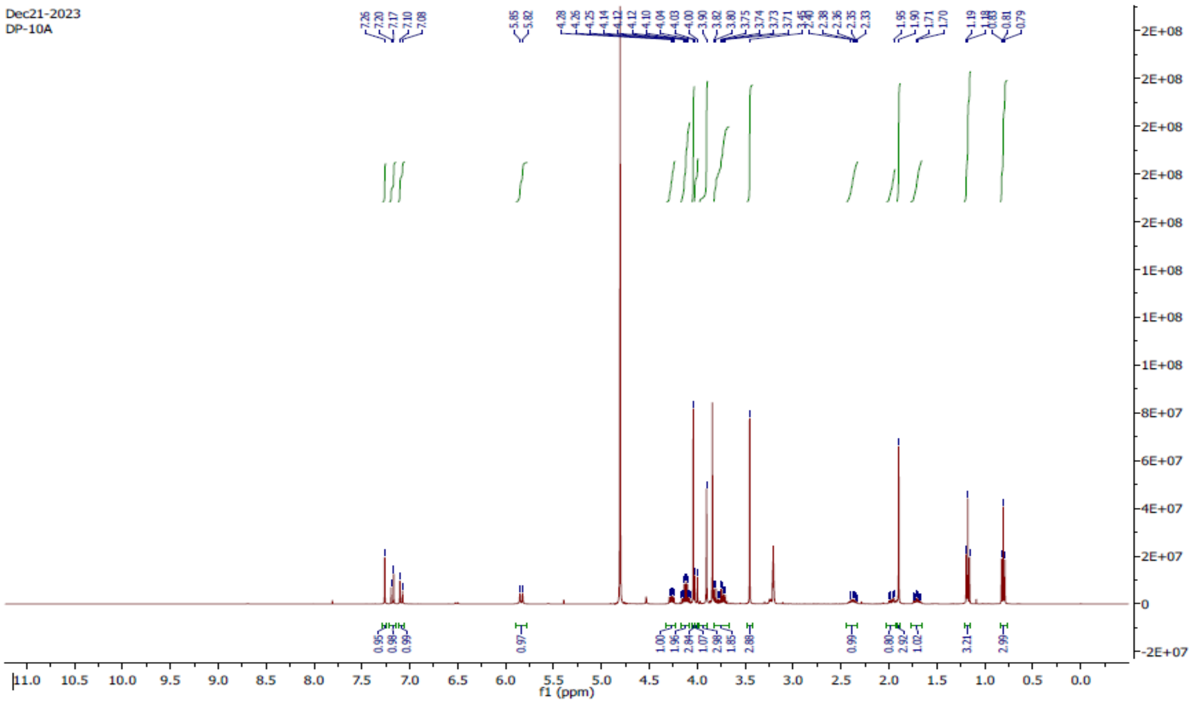

^{13}C NMR of 3h

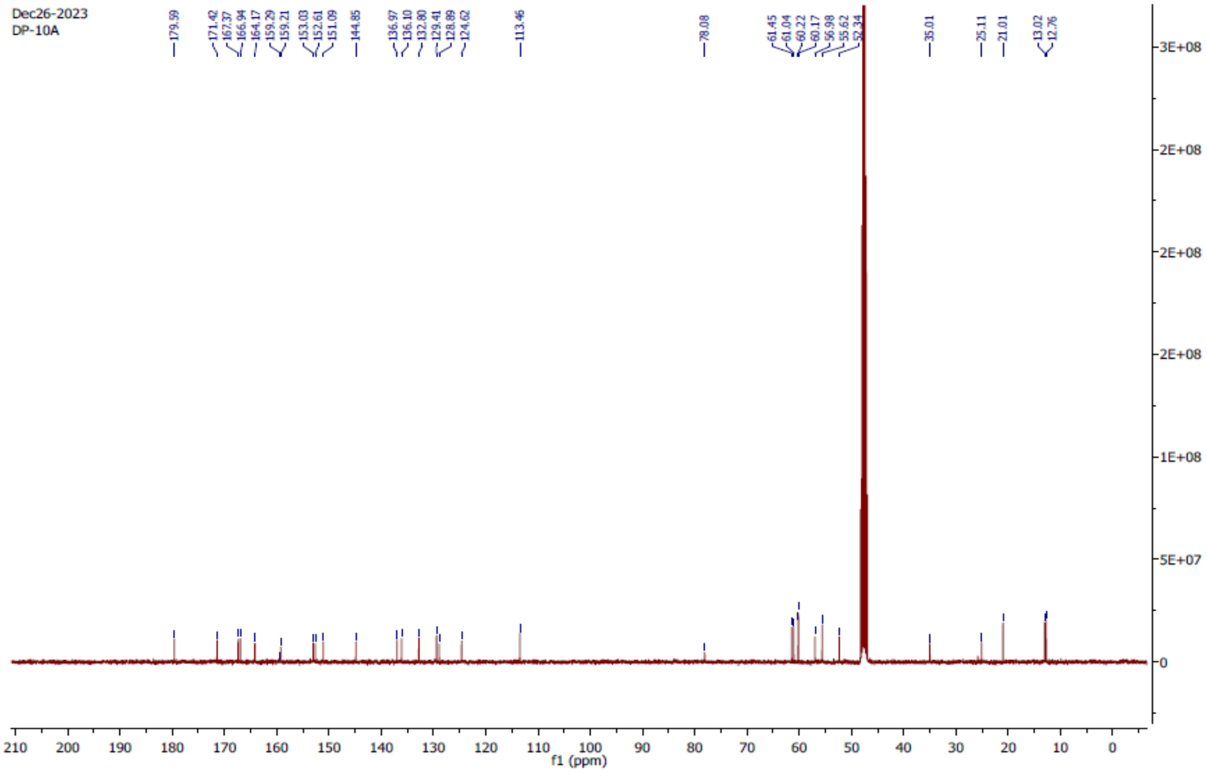

Dec26-2023
DP-10A
179.59
171.42
167.37
166.94
164.17
159.29
159.21
153.03
152.61
151.09
144.85
136.97
136.10
132.80
129.41
128.89
124.62
113.46
78.08
61.45
61.04
60.22
60.17
56.98
55.62
52.34
35.01
25.11
21.01
13.02
12.76
f1 (ppm)

Mass of 3i

Elemental Composition Report Page 1

Single Mass Analysis
Tolerance = 100.0 PPM / DBE: min = -1.5, max = 50.0
Element prediction: Off
Number of isotope peaks used for i-FIT = 3

Monoisotopic Mass, Even Electron Ions
44 formula(e) evaluated with 1 results within limits (up to 3 closest results for each mass)
Elements Used:
C: 0-31 H: 0-100 N: 0-3 O: 0-9

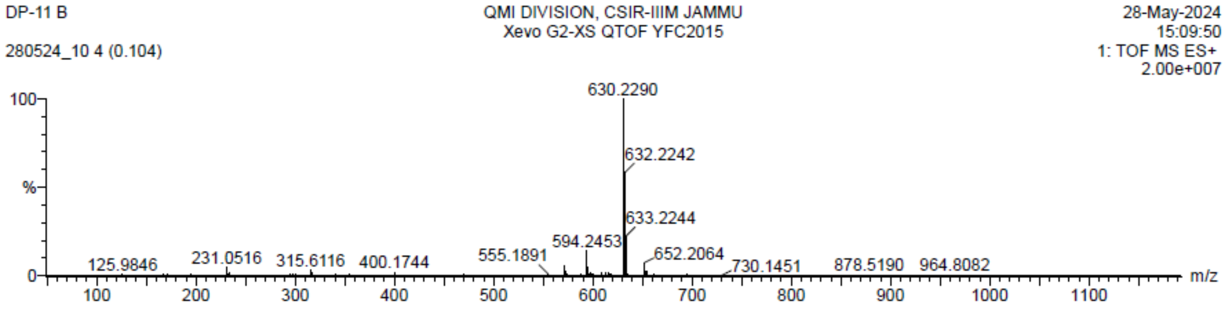

Minimum:				-1.5				
Maximum:		2.0	100.0	50.0				
Mass	Calc. Mass	mDa	PPM	DBE	i-FIT	Norm	Conf(%)	Formula
594.2453	594.2452	0.1	0.2	15.5	753.5	n/a	n/a	C31 H36 N3 O9

^{1}H NMR of 3i

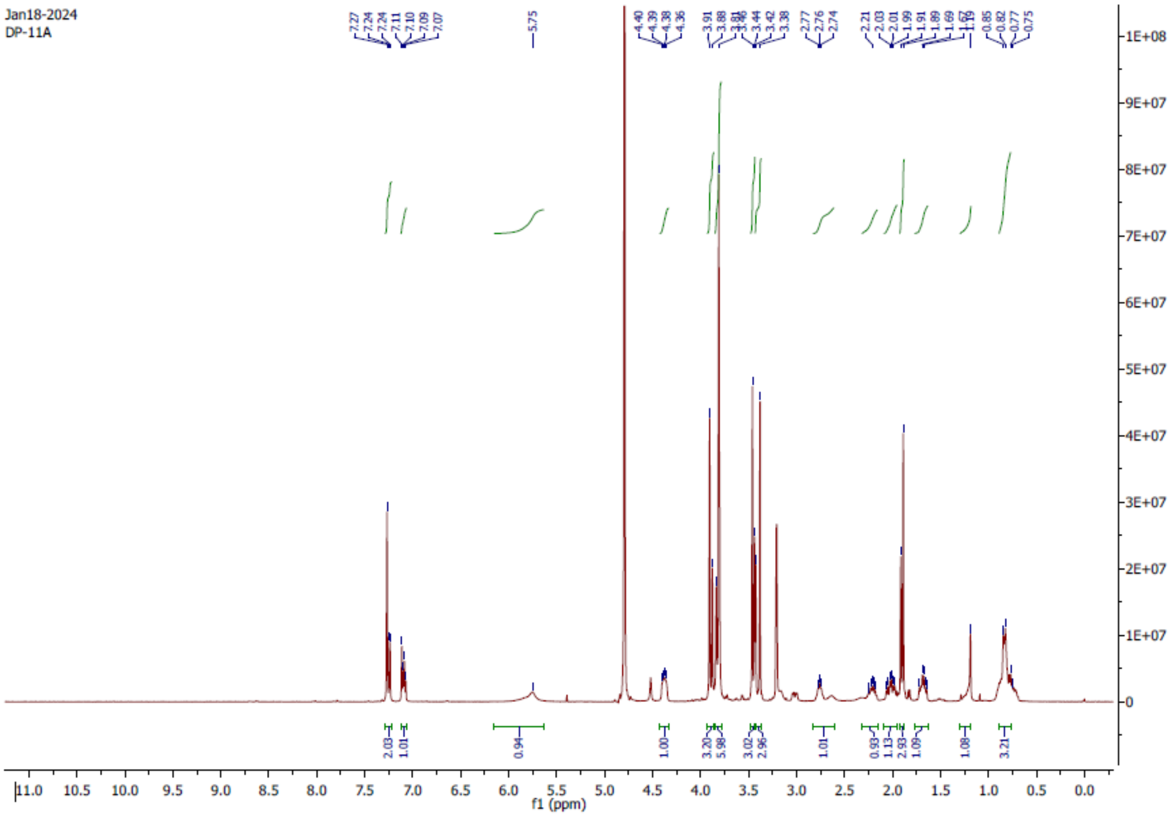

^{13}C NMR of 3i

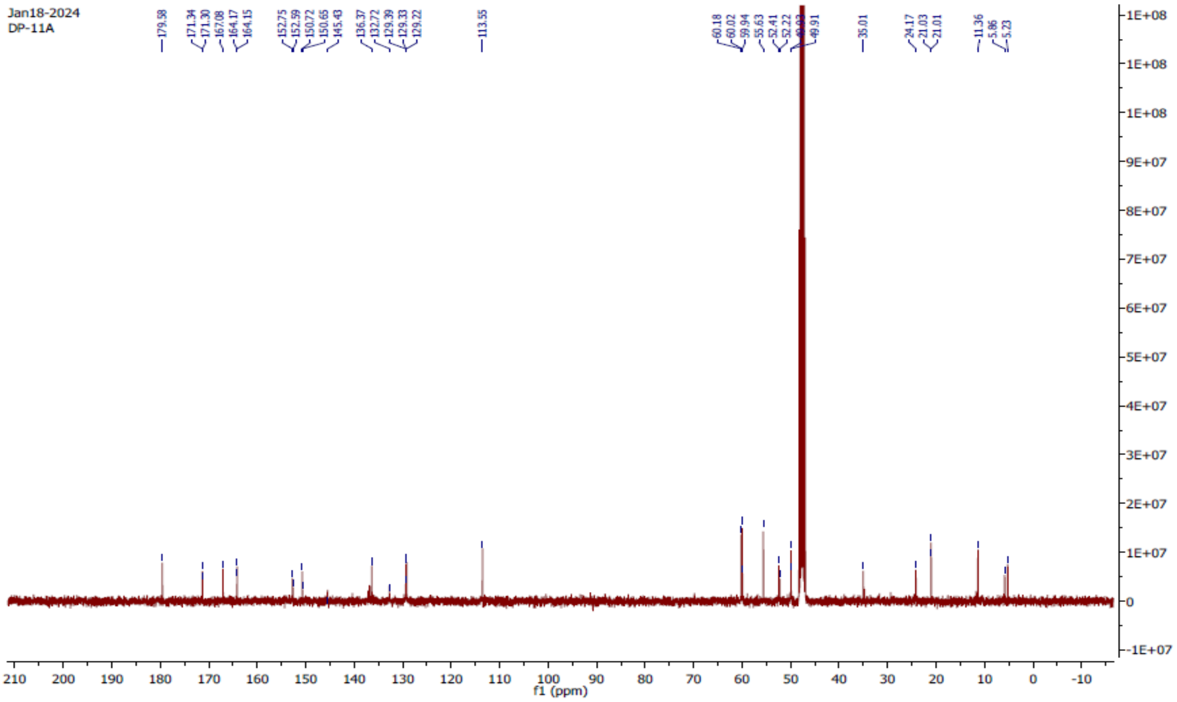

Mass of 3j

Sample Information

**Sample Name**	**: DP-11B**	**Sample ID**	**: DP-11B**
**Tray#**	**: 1**	**Vial#**	**: 17**
**Injection Volume**	**: 2**	**Data File**	**: 22-05-2024 -JSM_10.lcd**
**Method File**	**: MASS SCANNN 13APRIL2021.lcm**	**Processed by**	**: System Administrator**
**Date Processed**	**: 5/22/2024 12:54:15 PM**		

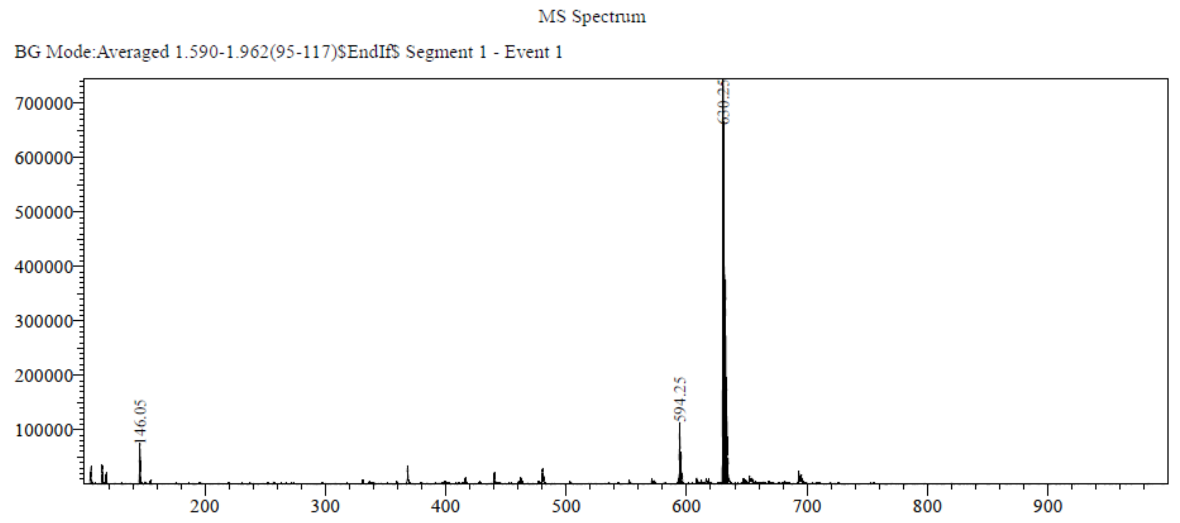

^{1}H NMR of 3j

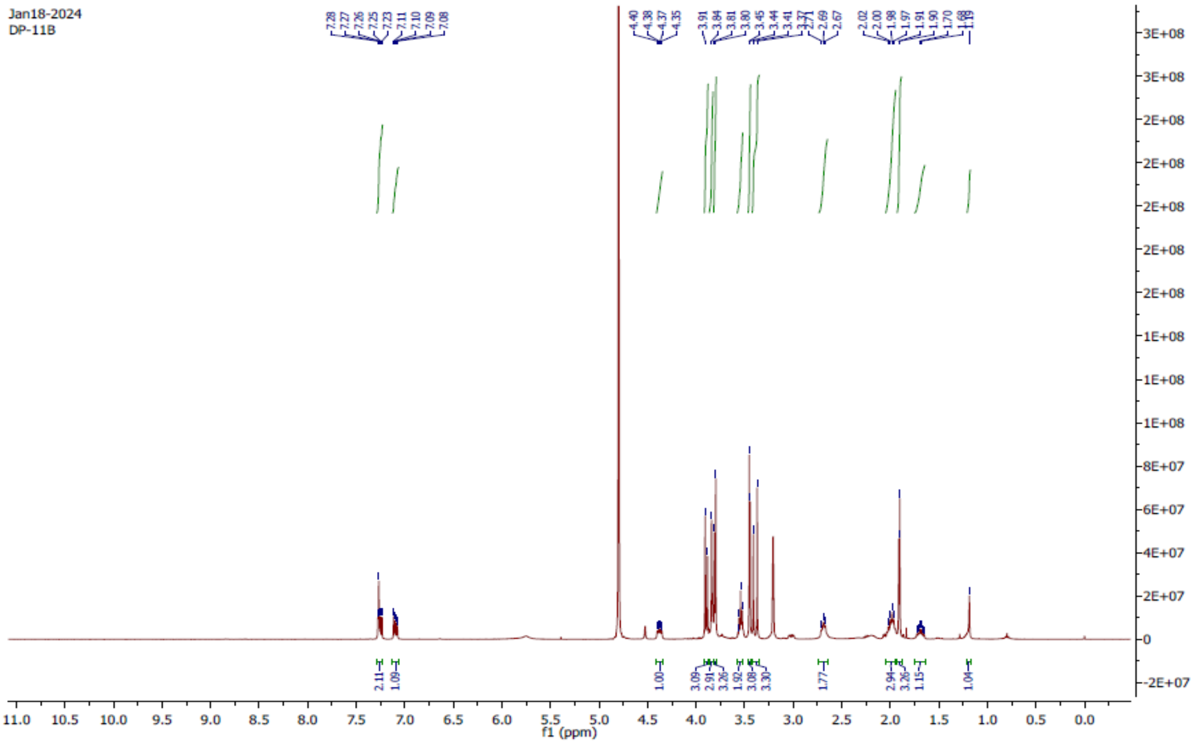

^{13}C NMR of 3j

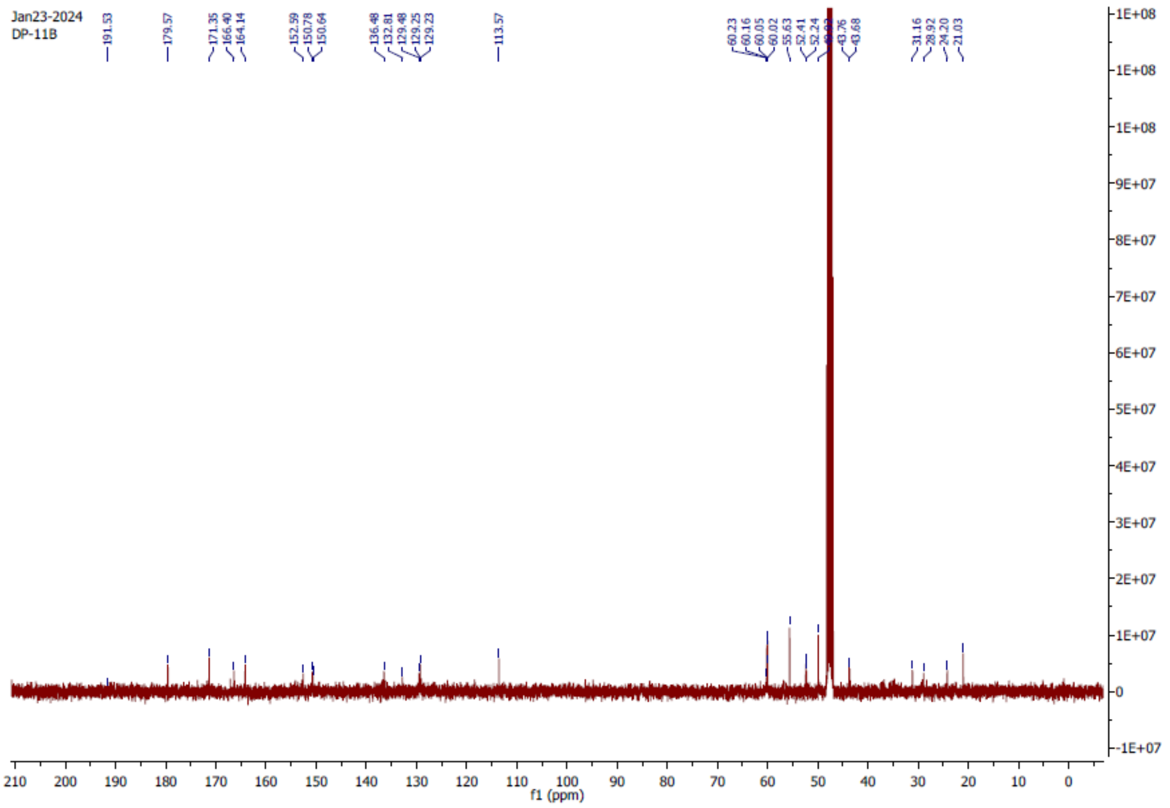

MTT ASSAY:

Table S1: Half-maximal inhibitory concentration (IC_{50}, µmol/L) of Colchicine and Paclitaxel (a standard compound for antiproliferative activity) was determined against various human cancer cell lines and a normal cell line using another MTT cytotoxicity assay.

**Cell line**	**Colchicine IC_{50} (µM)**	**Paclitaxel IC_{50} (µM)**
A549	0.21±0.02	0.028±0.008
A375	0.022±0.008	0.017±0.028
MCF-7	5.69±0.66	0.014±0.001
MDA-MB 231	4.11±0.8	0.020±0.002

Colchicine MTT references:

A549 =PMID: 32106039, PMID: 33465696, PMID: 36629423, PMID: 34801948

A375= PMID: 29180476, PMID: 31043459, PMID: 22410804

MCF-7 = PMID: 33588683, PMID: 38908129, PMID: 36067627

MDA-MB 231 = PMID: 38908129
